# Supplementary material for: Inference of cell type content from human brain transcriptomic datasets illuminates the effects of age, manner of death, dissection, and psychiatric diagnosis
Source: PLoS One. 2018 Jul 17;13(7):e0200003. doi: 10.1371/journal.pone.0200003 (PMC6049916; doi:10.1371/journal.pone.0200003)
Supplement: S1 Text — (PDF) [file pone.0200003.s001.pdf]

Supplementary material for:

Inference of Cell Type Content from Human Brain Transcriptomic Datasets Illuminates the Effects of Age, Manner of Death, Dissection, and Psychiatric Diagnosis

*Megan Hastings Hagenauer[1], Anton Schulmann[2], Jun Z. Li[3], Marquis P. Vawter[4], David M. Walsh[4], Robert C. Thompson[1], Cortney A. Turner[1], William E. Bunney[4], Richard M. Myers[5], Jack D. Barchas[6], Alan F. Schatzberg[7], Stanley J. Watson[1], Huda Akil[1]

[1]Mol. Behavioral Neurosci. Inst., Univ. of Michigan, Ann Arbor, MI, USA; [2] Janelia Research Campus, Howard Hughes Medical Institute, Ashburn, VA, USA, [3]Genet., Univ. of Michigan, Ann Arbor, MI, USA; [4]Univ. of California, Irvine, CA; [5]HudsonAlpha Inst. for Biotech., Huntsville, AL, USA; [6]Stanford, Palo Alto, CA, [7]Cornell, New York, NY, USA

*Corresponding Author: Megan Hastings Hagenauer

e-mail: hagenaue@umich.edu

Molecular Behavioral Neuroscience Institute (MBNI)

205 Zina Pitcher Pl.

Ann Arbor, MI 48109

Additional methods: Detailed preprocessing methods for the transcriptomic datasets

RNA-Seq data from purified cell types (GSE52564 and GSE67835)

As initial validation, we used our method to predict sample cell type identity in two RNA-Seq datasets derived from purified cell types: one derived from from purified cortical cell types in mice (n=17: two samples per cell type and 3 whole brain samples: GSE52564) [18], and one derived from 466 single-cells dissociated from freshly-resected human cortex (GSE67835) [2]. The RNA-Seq data that we downloaded from GEO was already in the format of FPKM values (Fragments Per Kilobase of exon model per million mapped fragments) [18] or counts per gene [2]. To stabilize the variance in the data, we used a log(2) transformation (after adding 1), and then filtered out the data for any genes that completely lacked variation across samples (sd=0; final gene count: GSE52564: 17148, GSE67835: 21627). Then we applied the methods now found in the BrainInABlender package, and examined the correlation between each of the cell type indices and sample cell type identity (excluding the fetal, whole brain, and "hybrid" cells). The code for these analyses can be found at https://github.com/hagenaue/CellTypeAnalyses_Darmanis and https://github.com/hagenaue/CellTypeAnalyses_Zhang.

Microarray data from artificial cell mixtures (GSE19380)

As further validation, we determined whether relative cell type balance could be accurately deciphered from microarray data for samples containing artificially-generated mixtures of cultured cells from the cerebral cortices of rat P1 pups (GSE19380, [12]). The microarray profiling was performed using a Affymetrix Rat Genome 230 2.0 Array. According to the methods on GEO, this data had already

undergone probeset summarization and normalization using robust multi-array averaging (RMA, *affy* package [87]), including background subtraction, summarization by median polish, log(2) transformation, and quantile normalization. We then predicted the cell content of each sample from the microarray data using BrainInABlender, and correlated these predictions with the actual percent of each cell type found in the mixtures (**Fig 5** in main text). The code for these analyses can be found at: https://github.com/hagenaue/CellTypeAnalyses_KuhnMixtures/tree/master.

Pritzker dorsolateral prefrontal cortex microarray dataset (GSE92538)

The original dataset included tissue from 172 high-quality human post-mortem brains donated to the Brain Donor Program at the University of California, Irvine with the consent of the next of kin. Frozen coronal slabs were macro-dissected to obtain dorsolateral prefrontal cortex samples. Clinical information was obtained from medical examiners, coroners' medical records, and a family member. Patients were diagnosed with either Major Depressive Disorder, Bipolar Disorder, or Schizophrenia by consensus based on criteria from the Diagnostic and Statistical Manual of Mental Disorders [88]. Due to the extended nature of this study, this sample collection occurred in waves ("cohorts") over a period of many years.

As described previously [28,62], total RNA from these samples was then distributed to laboratories at three different institutions (University of Michigan (UM), University of California-Davis (UCD), University of California-Irvine (UCI)) for hybridization to either Affymetrix HT-U133A or HT-U133Plus-v2 chips (1-5 replicates per sample, n=367). Before conducting the current analysis, the subset of probes found on both the Affymetrix HT-U133A and HT-U133Plus-v2 chips was extracted, reannotated for probe-to-transcript correspondance [89], summarized using RMA [87], log(2)-transformed, quantile normalized, and gender-checked. Then, 15 batches of highly-correlated samples were identified that were defined by a combination of cohort, chip, and laboratory (**Fig A**).

Batch#	Site	Chip	Cohort	Control	BP	MDD	SCHIZ
1	UCD	U133A	Dep Cohort 1 & 2	20	9	11	0
2	UCD	U133A	Dep Cohort 3	11	6	5	0
3	UCD	U133A	Dep Cohort 4	16	4	7	0
4	UCD	U133Plus2	Dep Cohort 5	13	5	10	0
5	UCD	U133A	Schiz Cohort 1	9	0	0	9
6	UCD	U133Plus2	Schiz Cohort 1	8	0	0	8
7	UCD	U133Plus2	Schiz Cohort 2	8	0	0	10
8	UCI	U133A	Schiz Cohort 1	9	0	0	9
9	UM	U133A	Dep Cohort 1	16	10	9	0
10	UM	U133A	Dep Cohort 2	3	2	5	0
11	UM	U133A	Dep Cohort 3 & 4	27	11	11	0
12	UM	U133Plus2	Dep Cohort 5	13	5	10	0
13	UM	U133Plus2	Dep Cohort 6	7	2	9	3
14	UM	U133A	Schiz Cohort 1	9	0	0	9
15	UM	U133Plus2	Schiz Cohort 2	9	0	0	10

Fig A. The number of microarray chips run in each batch, defined by processing site, Affymetrix chip type, and sample collection cohort. Samples from the four diagnostic categories (Control, Bipolar Disorder, Major Depressive Disorder, Schizophrenia) were unevenly distributed across batches.

Samples that exhibited markedly low average sample-sample correlation coefficients (<0.85: outliers) were removed from the dataset, including data from one batch that exhibited overall low sample-sample correlation coefficients with other batches and duplicate microarrays. The batch effects were then subtracted out using median-centering (detailed procedure: [62]) and the replicate samples were averaged for each subject. Our current analyses began with this sample-level summary gene expression data (GSE92538). We further removed data from any subjects lacking information regarding critical pre- or post-mortem variables necessary for our analysis (final sample size: n=157). The code for these analyses can be found at https://github.com/hagenaue/CellTypeAnalyses_PritzkerAffyDLPFC.

Allen Brain Atlas cross-regional microarray dataset

The Allen Brain Atlas microarray data was downloaded from http://human.brain-map.org/microarray/search in December 2015. This microarray survey was performed in brain-specific batches, with multiple batches per subject. To remove technical variation across batches, a variety of normalization procedures had been performed by the original authors both within and across batches using internal controls, as well as across subjects [90]. The dataset available for download had already

been log(2)-transformed and converted to z-scores using the average and standard deviation for each probe. These normalization procedures were designed to remove technical artifacts while best preserving cross-regional effects in the data, but the full information about relative levels of expression within an individual sample were unavailable and the effects of subject-level variables (such as age and pH) were likely to be de-emphasized due to the inability to fully separate out subject and batch during the normalization process. The 30,000 probes mapped onto 18,787 unique genes (as determined by gene symbol). The code for these analyses can be found at https://github.com/hagenaue/CellTypeAnalyses_AllenBrainAtlas.

Human cortical microarray dataset GSE53987 (described in Lanz et al. [30])

The full publicly-available dataset GSE53987 [30] contained Affymetrix U133Plus2 microarray data from 205 post-mortem human brain samples from three brain regions: the DLPFC (Brodmann Area 46, focusing on gray matter only (Lanz T.A., *personal communication*)), the hippocampus, and the striatum. These samples were collected by the University of Pittsburgh brain bank. For the purposes of our current analysis, we only downloaded the microarray .CEL files for the dorsolateral prefrontal cortex samples. We summarized these data with RMA [87] using a custom up-to-date chip definition file (.cdf) to define probe-to-transcript correspondence ("hgu133plus2hsentrezgcdf_19.0.0.tar.gz" from http://nmg-r.bioinformatics.nl/NuGO_R.html [89]). This process included background subtraction, log(2)-transformation, and quantile normalization. Gene Symbol annotation for probeset Entrez gene ids were provided by the R package *org.Hs.eg.db*. To control for technical variation, the sample processing batches were estimated using the microarray chip scan dates extracted from the .CEL files (using the function *protocolData* in the *GEOquery* package [91]), but all chips for the DLPFC were scanned on the same date. RNA degradation was estimated using the R package AffyRNADegradation [33]. During quality control, two samples were removed - GSM1304979 had a range of sample-sample correlations that was

unusually low compared (median=0.978) compared to the range for the dataset as a whole (median: 0.993) and GSM1304953 appeared to be falsely identified as female (signal for XIST<7). The code for these analyses can be found at:

https://github.com/hagenaue/CellTypeAnalyses_LanzHumanDLPFC/tree/master

Human cortical microarray dataset GSE21138 (described in Narayan et al. [32])

The publicly-available dataset GSE21138 [32]) contained Affymetrix U133Plus2 microarray data from 59 post-mortem human brain samples from the DLPFC (Brodmann Area 46, gray matter only (Thomas E.A., *personal communication*)) collected by the Mental Health Research Institute in Victoria, Australia. The procedures for data download and pre-processing were identical to those used above for GSE53987 with a few minor exceptions. In particular, there were six separate scan dates associated with the microarray .CEL files, but one of these scan dates was not included as a co-variate in our analyses because it had an n=1 ("06/14/06"). During quality control, the data for two subjects were removed because they appeared to be falsely-identified as male (XIST>7, GSM528839 & GSM528840) or falsely-identified as female (XIST<7, GSM528880). Data for two more subjects were removed as outliers due to having an unusually low range of sample-sample correlations (GSM528866, GSM528873) as compared to the dataset as a whole. The code for these analyses can be found at: https://github.com/hagenaue/CellTypeAnalyses_NarayanHumanDLPFC.

Human cortical microarray dataset GSE21935 (described in Barnes et al. [31])

The publicly-available dataset GSE21935 [31] contained Affymetrix U133Plus2 microarray data from 42 post-mortem human brain samples from the temporal cortex (Brodmann Area 22) collected at the

Charing Cross campus of the Imperial College of London. The procedures for data download and pre-processing were identical to those used above for GSE53987 with a few minor exceptions. In particular, there were two separate scan dates associated with the microarray .CEL files, but they were closely spaced (6/25/04 vs. 6/29/04) and we did not find any strong association between scan date and the top principal components of variation in the data, so we opted to not include scan date as a co-variate in our statistical models. Quality control did not identify any problematic samples. The code for these analyses can be found at:
https://github.com/hagenaue/CellTypeAnalyses_BarnesHumanCortex/tree/master.

CommonMind Consortium human cortical RNA-Seq dataset (described in Fromer et al. [34])

The CommonMind Consortium (CMC) RNA-seq dataset profiled prefrontal cortex samples from 603 individuals [34] collected at three brain banks: Mount Sinai School of Medicine, University of Pittsburgh, and University of Pennsylvania. This dataset was downloaded as GRCh37-aligned bam files from the CommonMind Consortium Knowledge Portal (https://www.synapse.org/CMC). Tophat-aligned bam files were converted back to fastq format and mapped to GRCh38 using HISAT2 [92] with default settings. Reads mapping uniquely to exons were then counted using subread featureCounts with ensembl transcript models. Cell type indices were calculated using logCPM values, and analysis of differential gene expression was performed using using the limma/voom method [93] with observed precision weights in a weighted least squares linear regression. Prior to upload, poor quality samples from the original dataset [34] had already been removed (<50 million reads, RIN<5.5) and replaced with higher quality samples. We further excluded data from 10 replicates and 89 individuals with incomplete demographic data (missing pH; final sample size: n=514). The dataset was further filtered using an expression threshold (CPM>1 in at least 50 individuals) which reduced the dataset to data from around 17,000 genes. The code for these analyses can be found at https://github.com/aschulmann/CMC_celltype_index.

Additional validation for the BrainInABlender method

BrainInABlender can predict relative cell content in datasets from purified cells and *In Silico* mixtures

As initial validation, we used the BrainInABlender method to predict the relative balance of cell types in samples with known cell content (purified cells and artificial cell mixtures). To do this analysis, we used two RNA-Seq datasets: one derived from from purified cortical cell types in mice (GSE52564) [18], and one derived from human single-cell RNA-Seq (GSE67835) [2]. In general, we found that the statistical cell type indices easily predicted the cell type identities of the original samples (**Fig B).** The correlation between each of the cell type indices and their respective cell type was very strong within the mouse purified, pooled cell type dataset (R^2 between 0.41-0.90) and moderate in the noisier human single-cell dataset (R^2 between 0.14-0.67), but typically still much higher than the correlation with other cell types. This was true regardless of the publication from which the cell type specific genes were derived: cell type specific gene lists derived from publications using different species (human vs. mouse), platforms (microarray vs. RNA-Seq), methodologies (florescent cell sorting vs. suspension), or statistical stringency all performed fairly equivalently, with some minor exception. Notably, the cell type indices derived from the cell type specific gene lists in Doyle et al. ([15], originally identified using TRAP methodology) tended to perform poorly. In both validation datasets, the cell type index Oligodendrocyte_All_Doyle_Cell_2008 did not properly predict the cell identity of the samples, and Neuron_Neuron_CCK_Doyle_Cell_2008 and Neuron_Interneuron_CORT_Doyle_Cell_2008 were elevated in non-neuronal cell types. Later, we found that other cell type specific gene lists from the Doyle et al. study [15] included a high percentage of genes that appeared non-specific to their respective cell type (**Fig C**; Neuron_CorticoSpinal_Doyle_Cell_2008, Neuron_CorticoStriatal_Doyle_Cell_2008),

leading us to remove all cell type specific gene lists derived from this publication from later versions of BrainInABlender, although we found that it did not dramatically alter our results. In general, the cell type indices associated with immature oligodendrocytes were also somewhat inconsistent. For example, neither of the immature oligodendrocyte cell type indices derived from gene lists in Zhang et al. [18] could predict OPC sample cell identity in the human single cell dataset [2] (R^2<0.02), perhaps due to differences in developmental stage and culture conditions. As would be expected, the cell type indices derived from cell type specific genes identified by the same publication that produced the test datasets [2,18] were (by definition) superb predictors of the sample cell identity in their own dataset, and were thus excluded from later validation analyses.

	Zhang_Astrocyte	Zhang_Endothelial	Zhang_Microglia	Zhang_Neuron	Zhang_Oligodendrocyte	Zhang_NFO	Zhang_OPC	Darmanis_Astrocyte	Darmanis_Endothelial	Darmanis_Microglia	Darmanis_Neuron	Darmanis_Oligodendrocyte	Darmanis_OPC
Astrocyte_All_Cahoy_JNeuro_2008	0.84	0.09	0.04	0.00	0.05	0.09	0.03	0.67	0.01	0.04	0.08	0.08	0.01
Astrocyte_All_Darmanis_PNAS_2015	0.67	0.12	0.04	0.02	0.12	0.10	0.07	0.87	0.02	0.02	0.17	0.05	0.02
Astrocyte_All_Doyle_Cell_2008	0.77	0.10	0.02	0.01	0.10	0.07	0.07	0.48	0.04	0.01	0.09	0.02	0.01
Astrocyte_All_Zeisel_Science_2015	0.74	0.09	0.09	0.01	0.12	0.01	0.09	0.25	0.04	0.11	0.00	0.04	0.02
Astrocyte_All_Zhang_JNeuro_2014	0.90	0.08	0.06	0.00	0.05	0.07	0.00	0.28	0.01	0.05	0.00	0.10	0.02
Endothelial_All_Daneman_PLOS_2010	0.02	0.99	0.02	0.02	0.05	0.04	0.02	0.01	0.27	0.01	0.00	0.02	0.01
Endothelial_All_Darmanis_PNAS_2015	0.01	0.90	0.00	0.09	0.08	0.09	0.00	0.01	0.84	0.00	0.05	0.01	0.00
Endothelial_All_Zeisel_Science_2015	0.00	0.90	0.02	0.01	0.19	0.04	0.00	0.00	0.14	0.03	0.01	0.02	0.03
Endothelial_All_Zhang_JNeuro_2014	0.02	0.99	0.03	0.02	0.05	0.04	0.02	0.03	0.25	0.02	0.00	0.00	0.01
Microglia_All_Darmanis_PNAS_2015	0.08	0.03	0.88	0.05	0.02	0.06	0.02	0.02	0.01	0.70	0.06	0.01	0.04
Microglia_All_Zeisel_Science_2015	0.03	0.01	0.87	0.05	0.13	0.05	0.02	0.02	0.00	0.30	0.00	0.04	0.01
Microglia_All_Zhang_JNeuro_2014	0.05	0.02	0.95	0.05	0.04	0.05	0.00	0.01	0.00	0.48	0.04	0.02	0.01
Neuron_All_Cahoy_JNeuro_2008	0.06	0.06	0.05	0.87	0.03	0.05	0.03	0.06	0.07	0.09	0.64	0.10	0.04
Neuron_All_Darmanis_PNAS_2015	0.03	0.14	0.06	0.74	0.03	0.04	0.10	0.18	0.05	0.08	0.79	0.08	0.03
Neuron_All_Zhang_JNeuro_2014	0.02	0.04	0.03	0.99	0.03	0.04	0.01	0.08	0.03	0.06	0.37	0.02	0.04
Neuron_CorticoSpinal_Doyle_Cell_2008	0.01	0.01	0.23	0.61	0.20	0.01	0.07	0.02	0.01	0.06	0.17	0.03	0.01
Neuron_CorticoStriatal_Doyle_Cell_2008	0.00	0.01	0.11	0.40	0.14	0.29	0.00	0.01	0.00	0.00	0.03	0.01	0.00
Neuron_CorticoThalamic_Doyle_Cell_2008	0.01	0.12	0.02	0.62	0.25	0.11	0.03	0.03	0.01	0.00	0.20	0.10	0.00
Neuron_Glutamate_Sugino_NatNeuro_2006	0.06	0.03	0.07	0.38	0.07	0.03	0.26	0.04	0.01	0.07	0.25	0.02	0.02
Neuron_Pyramidal_Cortical_Zeisel_Science_2015	0.02	0.06	0.04	0.70	0.14	0.03	0.12	0.04	0.04	0.06	0.39	0.06	0.03
Neuron_GABA_Sugino_NatNeuro_2006	0.01	0.01	0.27	0.49	0.23	0.01	0.13	0.04	0.05	0.09	0.40	0.04	0.02
Neuron_Interneuron_CORT_Doyle_Cell_2008	0.02	0.01	0.12	0.49	0.40	0.01	0.09	0.02	0.02	0.05	0.17	0.01	0.02
Neuron_Interneuron_Zeisel_Science_2015	0.00	0.10	0.11	0.68	0.15	0.00	0.11	0.06	0.06	0.09	0.49	0.04	0.04
Neuron_Neuron_CCK_Doyle_Cell_2008	0.02	0.54	0.05	0.00	0.12	0.19	0.11	0.04	0.01	0.01	0.09	0.00	0.01
Neuron_Neuron_PNOC_Doyle_Cell_2008	0.05	0.03	0.01	0.97	0.04	0.07	0.00	0.07	0.04	0.05	0.41	0.03	0.05
Oligodendrocyte_All_Cahoy_JNeuro_2008	0.17	0.08	0.03	0.12	0.41	0.36	0.00	0.04	0.00	0.01	0.11	0.60	0.00
Oligodendrocyte_All_Doyle_Cell_2008	0.02	0.00	0.22	0.09	0.15	0.05	0.61	0.00	0.01	0.05	0.06	0.01	0.01
Oligodendrocyte_All_Zeisel_Science_2015	0.05	0.07	0.18	0.10	0.41	0.34	0.00	0.01	0.03	0.10	0.00	0.21	0.03
Oligodendrocyte_Mature_Darmanis_PNAS_2015	0.02	0.17	0.09	0.12	0.48	0.26	0.00	0.04	0.00	0.00	0.13	0.77	0.00
Oligodendrocyte_Mature_Doyle_Cell_2008	0.13	0.05	0.16	0.07	0.52	0.23	0.00	0.04	0.02	0.02	0.05	0.29	0.00
Oligodendrocyte_Myelinating_Zhang_JNeuro_2014	0.13	0.04	0.06	0.06	0.67	0.17	0.03	0.02	0.00	0.01	0.07	0.48	0.01
Oligodendrocyte_Newly-Formed_Zhang_JNeuro_2014	0.05	0.08	0.28	0.00	0.02	0.70	0.02	0.05	0.02	0.08	0.26	0.01	0.02
Oligodendrocyte_Progenitor Cell_Darmanis_PNAS_2015	0.01	0.10	0.05	0.01	0.22	0.14	0.63	0.03	0.00	0.00	0.03	0.01	0.68
Oligodendrocyte_Progenitor Cell_Zhang_JNeuro_2014	0.02	0.12	0.15	0.02	0.14	0.08	0.64	0.05	0.02	0.08	0.21	0.04	0.01

Fig B. Cell type indices successfully predict sample cell type in purified cell type RNA-Seq data. Cell type indices derived from cell type specific transcripts originating from publications using different species, methodologies, and platforms could successfully predict the sample cell types within two RNA-Seq datasets (mouse purified cells (Zhang et al. [18]) and human single cells (Darmanis et al. [2]). Depicted in the table are the R^2 values indicating how much of the variance within any particular cell type index (row) is explained by a particular sample cell type (column, NFO: "newly-formed oligodendrocyte"). The cell type indices are named after their origin (primary cell type, subtype, and publication), and the primary cell type category is further identified by color (lavender: astrocytes, orange: endothelial, green: microglia, yellow: mural, purple: neuron_all, blue: neuron_projection, red: neuron_interneuron, pink: oligodendrocyte, white: oligodendrocyte progenitor cell (OPC)).

For further analyses, individual cell type indices were averaged within each of the primary cell type categories to produce ten consolidated primary cell-type indices for each sample. To perform this consolidation, we also removed any transcripts that were identified as "cell type specific" to multiple primary cell type categories (**Fig C**).

A.

Reference Publications: Cell Type Specific Gene Lists	# Genes	Genes (Specific)	% Specific
Astrocyte_All_Cahoy_JNeuro_2008	73	64	88%
Astrocyte_All_Darmanis_PNAS_2015	21	20	95%
Astrocyte_All_Doyle_Cell_2008	25	25	100%
Astrocyte_All_Zeisel_Science_2015	240	216	90%
Astrocyte_All_Zhang_JNeuro_2014	40	32	80%
Endothelial_All_Daneman_PLOS_2010	49	43	88%
Endothelial_All_Darmanis_PNAS_2015	21	19	90%
Endothelial_All_Zeisel_Science_2015	353	319	90%
Endothelial_All_Zhang_JNeuro_2014	40	30	75%
Microglia_All_Darmanis_PNAS_2015	21	19	90%
Microglia_All_Zeisel_Science_2015	436	396	91%
Microglia_All_Zhang_JNeuro_2014	40	37	93%
Mural_All_Zeisel_Science_2015	155	146	94%
Mural_Pericyte_Zhang_JNeuro_2014	40	28	70%
Mural_Vascular_Daneman_PLOS_2010	50	33	66%
Neuron_All_Cahoy_JNeuro_2008	80	57	71%
Neuron_All_Darmanis_PNAS_2015	21	16	76%
Neuron_All_Zhang_JNeuro_2014	40	27	68%
Neuron_CorticoSpinal_Doyle_Cell_2008	25	16	64%
Neuron_CorticoStriatal_Doyle_Cell_2008	25	9	36%
Neuron_CorticoThalamic_Doyle_Cell_2008	25	15	60%
Neuron_Glutamate_Sugino_NatNeuro_2006	67	59	88%
Neuron_Pyramidal_Cortical_Zeisel_Science_2015	294	258	88%
Neuron_GABA_Sugino_NatNeuro_2006	32	28	88%
Neuron_Interneuron_CORT_Doyle_Cell_2008	25	20	80%
Neuron_Interneuron_Zeisel_Science_2015	365	328	90%
Neuron_Neuron_CCK_Doyle_Cell_2008	25	18	72%
Neuron_Neuron_PNOC_Doyle_Cell_2008	24	17	71%
Oligodendrocyte_All_Cahoy_JNeuro_2008	50	48	96%
Oligodendrocyte_All_Doyle_Cell_2008	25	20	80%
Oligodendrocyte_All_Zeisel_Science_2015	453	421	93%
Oligodendrocyte_Mature_Darmanis_PNAS_2015	21	20	95%
Oligodendrocyte_Mature_Doyle_Cell_2008	25	19	76%
Oligodendrocyte_Myelinating_Zhang_JNeuro_2014	40	40	100%
Oligodendrocyte_Newly-Formed_Zhang_JNeuro_2014	39	25	64%
Oligodendrocyte_Progenitor Cell_Darmanis_PNAS_2015	21	12	57%
Oligodendrocyte_Progenitor Cell_Zhang_JNeuro_2014	40	26	65%
RBC_All_GeneCardSearch_Hemoglobin_ErythrocyteSpecific	17	10	59%

B.

	Astrocyte_All_Cahoy_JNeuro_2008	Astrocyte_All_Darmanis_PNAS_2015	Astrocyte_All_Doyle_Cell_2008	Astrocyte_All_Zeisel_Science_2015	Astrocyte_All_Zhang_JNeuro_2014	Endothelial_All_Daneman_PLOS_2010	Endothelial_All_Darmanis_PNAS_2015	Endothelial_All_Zeisel_Science_2015	Endothelial_All_Zhang_JNeuro_2014	Microglia_All_Darmanis_PNAS_2015	Microglia_All_Zeisel_Science_2015	Microglia_All_Zhang_JNeuro_2014	Mural_All_Zeisel_Science_2015	Mural_Pericyte_Zhang_JNeuro_2014	Mural_Vascular_Daneman_PLOS_2010	Neuron_All_Cahoy_JNeuro_2008	Neuron_All_Darmanis_PNAS_2015	Neuron_All_Zhang_JNeuro_2014	Neuron_CorticoSpinal_Doyle_Cell_2008
Astrocyte_All_Cahoy_JNeuro_2008	100%	10%	8%	21%	23%	0%	0%	3%	0%	0%	3%	1%	0%	1%	4%	0%	0%	0%	0%
Astrocyte_All_Darmanis_PNAS_2015	33%	100%	0%	14%	5%	0%	0%	0%	0%	0%	0%	0%	0%	5%	0%	0%	0%	0%	0%
Astrocyte_All_Doyle_Cell_2008	24%	0%	100%	12%	0%	0%	0%	0%	0%	0%	0%	0%	0%	0%	0%	0%	0%	0%	0%
Astrocyte_All_Zeisel_Science_2015	6%	1%	1%	100%	3%	0%	0%	1%	0%	0%	3%	0%	0%	2%	0%	0%	0%	0%	0%
Astrocyte_All_Zhang_JNeuro_2014	43%	3%	0%	18%	100%	0%	0%	0%	0%	0%	0%	0%	0%	3%	10%	0%	0%	0%	0%
Endothelial_All_Daneman_PLOS_2010	0%	0%	0%	0%	0%	100%	2%	59%	10%	0%	2%	0%	2%	0%	0%	0%	0%	0%	6%
Endothelial_All_Darmanis_PNAS_2015	0%	0%	0%	0%	0%	5%	100%	5%	5%	0%	0%	0%	5%	0%	5%	0%	0%	0%	0%
Endothelial_All_Zeisel_Science_2015	1%	0%	0%	1%	0%	8%	0%	100%	8%	0%	2%	0%	0%	0%	1%	0%	0%	0%	3%
Endothelial_All_Zhang_JNeuro_2014	0%	0%	0%	0%	0%	13%	3%	70%	100%	0%	0%	3%	0%	0%	0%	3%	0%	0%	18%
Microglia_All_Darmanis_PNAS_2015	0%	0%	0%	0%	0%	0%	0%	0%	0%	100%	62%	19%	0%	5%	0%	0%	0%	0%	0%
Microglia_All_Zeisel_Science_2015	1%	0%	0%	2%	0%	0%	0%	2%	0%	3%	100%	7%	0%	1%	1%	0%	0%	0%	0%
Microglia_All_Zhang_JNeuro_2014	3%	0%	0%	0%	0%	0%	0%	3%	3%	10%	68%	100%	0%	3%	0%	0%	0%	0%	0%
Mural_All_Zeisel_Science_2015	0%	0%	0%	1%	0%	1%	1%	1%	0%	0%	1%	0%	100%	3%	2%	0%	0%	0%	0%
Mural_Pericyte_Zhang_JNeuro_2014	3%	3%	0%	15%	3%	0%	0%	3%	0%	3%	3%	3%	10%	100%	10%	0%	0%	0%	0%
Mural_Vascular_Daneman_PLOS_2010	6%	0%	0%	2%	8%	0%	2%	2%	0%	0%	6%	0%	6%	8%	100%	2%	0%	0%	0%
Neuron_All_Cahoy_JNeuro_2008	0%	0%	0%	0%	0%	0%	0%	1%	1%	0%	1%	0%	0%	0%	1%	100%	13%	9%	1%
Neuron_All_Darmanis_PNAS_2015	0%	0%	0%	5%	0%	0%	0%	0%	0%	0%	0%	0%	0%	0%	0%	43%	100%	10%	0%
Neuron_All_Zhang_JNeuro_2014	0%	0%	0%	0%	0%	0%	0%	0%	0%	0%	0%	0%	0%	0%	0%	18%	5%	100%	0%
Neuron_CorticoSpinal_Doyle_Cell_2008	0%	0%	0%	0%	0%	12%	0%	28%	20%	0%	0%	0%	0%	0%	0%	8%	0%	0%	100%
Neuron_CorticoStriatal_Doyle_Cell_2008	0%	0%	0%	0%	0%	4%	0%	4%	0%	4%	52%	4%	4%	8%	12%	0%	0%	0%	0%
Neuron_CorticoThalamic_Doyle_Cell_2008	0%	0%	0%	0%	0%	0%	0%	8%	0%	0%	0%	0%	0%	0%	0%	16%	0%	0%	4%
Neuron_Glutamate_Sugino_NatNeuro_2006	0%	0%	0%	0%	0%	0%	0%	3%	0%	0%	1%	0%	0%	0%	0%	3%	0%	0%	0%
Neuron_Pyramidal_Cortical_Zeisel_Science_2015	1%	0%	0%	1%	1%	0%	0%	1%	0%	0%	1%	0%	0%	0%	1%	2%	0%	1%	1%
Neuron_GABA_Sugino_NatNeuro_2006	0%	0%	0%	3%	0%	0%	0%	3%	0%	0%	0%	0%	0%	0%	0%	0%	0%	3%	0%
Neuron_Interneuron_CORT_Doyle_Cell_2008	0%	0%	0%	0%	0%	4%	0%	0%	4%	0%	0%	0%	4%	0%	0%	0%	0%	4%	0%
Neuron_Interneuron_Zeisel_Science_2015	1%	0%	0%	1%	0%	0%	0%	0%	0%	0%	1%	0%	0%	0%	0%	1%	1%	3%	0%
Neuron_Neuron_CCK_Doyle_Cell_2008	0%	0%	0%	0%	0%	4%	0%	4%	0%	0%	4%	0%	0%	0%	0%	4%	0%	0%	0%
Neuron_Neuron_PNOC_Doyle_Cell_2008	0%	0%	0%	0%	0%	0%	0%	0%	0%	0%	0%	0%	4%	0%	0%	17%	0%	8%	0%
Oligodendrocyte_All_Cahoy_JNeuro_2008	0%	0%	0%	2%	0%	0%	0%	0%	0%	0%	2%	0%	0%	0%	0%	0%	0%	0%	0%
Oligodendrocyte_All_Doyle_Cell_2008	0%	0%	0%	4%	0%	0%	0%	0%	0%	0%	4%	0%	0%	0%	4%	0%	0%	0%	0%
Oligodendrocyte_All_Zeisel_Science_2015	0%	0%	0%	2%	0%	0%	0%	0%	0%	0%	2%	0%	0%	0%	0%	0%	0%	0%	0%
Oligodendrocyte_Mature_Darmanis_PNAS_2015	0%	0%	0%	0%	0%	0%	0%	0%	0%	0%	5%	0%	0%	0%	0%	0%	0%	0%	0%
Oligodendrocyte_Mature_Doyle_Cell_2008	0%	0%	0%	8%	0%	0%	0%	4%	0%	0%	8%	0%	4%	0%	4%	0%	0%	0%	0%
Oligodendrocyte_Myelinating_Zhang_JNeuro_2014	0%	0%	0%	0%	0%	0%	0%	0%	0%	0%	0%	0%	0%	0%	0%	0%	0%	0%	0%
Oligodendrocyte_Newly-Formed_Zhang_JNeuro_2014	0%	0%	0%	3%	0%	0%	0%	3%	0%	0%	3%	0%	0%	0%	0%	3%	0%	0%	0%
Oligodendrocyte_Progenitor Cell_Darmanis_PNAS_2015	5%	0%	0%	5%	5%	0%	0%	5%	0%	0%	0%	0%	10%	0%	5%	0%	0%	0%	0%
Oligodendrocyte_Progenitor Cell_Zhang_JNeuro_2014	0%	0%	0%	3%	0%	0%	0%	5%	0%	0%	0%	0%	3%	5%	3%	0%	0%	0%	0%
RBC_All_GeneCardSearch_Hemoglobin_ErythrocyteSpecific	0%	0%	0%	6%	0%	0%	0%	18%	0%	0%	0%	0%	0%	0%	0%	0%	0%	0%	0%

	Neuron_CorticoStriatal_Doyle_Cell_2008	Neuron_CorticoThalamic_Doyle_Cell_2008	Neuron_Glutamate_Sugino_NatNeuro_2006	Neuron_Pyramidal_Cortical_Zeisel_Science_2015	Neuron_GABA_Sugino_NatNeuro_2006	Neuron_Interneuron_CORT_Doyle_Cell_2008	Neuron_Interneuron_Zeisel_Science_2015	Neuron_Neuron_CCK_Doyle_Cell_2008	Neuron_Neuron_PNOC_Doyle_Cell_2008	Oligodendrocyte_All_Cahoy_JNeuro_2008	Oligodendrocyte_All_Doyle_Cell_2008	Oligodendrocyte_All_Zeisel_Science_2015	Oligodendrocyte_Mature_Darmanis_PNAS_2015	Oligodendrocyte_Mature_Doyle_Cell_2008	Oligodendrocyte_Myelinating_Zhang_JNeuro_2014	Oligodendrocyte_Newly-Formed_Zhang_JNeuro_2014	Oligodendrocyte_Progenitor Cell_Darmanis_PNAS_2015	Oligodendrocyte_Progenitor Cell_Zhang_JNeuro_2014	RBC_All_GeneCardSearch_Hemoglobin_ErythrocyteSpecific
Astrocyte_All_Cahoy_JNeuro_2008	0%	0%	0%	3%	0%	0%	3%	0%	0%	0%	0%	0%	0%	0%	0%	0%	1%	0%	0%
Astrocyte_All_Darmanis_PNAS_2015	0%	0%	0%	0%	0%	0%	0%	0%	0%	0%	0%	0%	0%	0%	0%	0%	0%	0%	0%
Astrocyte_All_Doyle_Cell_2008	0%	0%	0%	0%	0%	0%	0%	0%	0%	0%	0%	0%	0%	0%	0%	0%	0%	0%	0%
Astrocyte_All_Zeisel_Science_2015	0%	0%	0%	1%	0%	0%	1%	0%	0%	0%	0%	3%	0%	1%	0%	0%	0%	0%	0%
Astrocyte_All_Zhang_JNeuro_2014	0%	0%	0%	5%	0%	0%	3%	0%	0%	0%	0%	0%	0%	0%	0%	0%	3%	0%	0%
Endothelial_All_Daneman_PLOS_2010	2%	0%	0%	0%	0%	2%	0%	2%	0%	0%	0%	0%	0%	0%	0%	0%	0%	0%	0%
Endothelial_All_Darmanis_PNAS_2015	0%	0%	0%	0%	0%	0%	0%	0%	0%	0%	0%	0%	0%	0%	0%	0%	0%	0%	0%
Endothelial_All_Zeisel_Science_2015	0%	0%	1%	1%	0%	0%	0%	0%	0%	0%	0%	0%	0%	0%	0%	0%	0%	1%	0%
Endothelial_All_Zhang_JNeuro_2014	0%	0%	0%	0%	0%	3%	0%	0%	0%	0%	0%	0%	0%	0%	0%	0%	0%	0%	0%
Microglia_All_Darmanis_PNAS_2015	5%	0%	0%	0%	0%	0%	0%	0%	0%	0%	0%	0%	0%	0%	0%	0%	0%	0%	0%
Microglia_All_Zeisel_Science_2015	3%	0%	0%	1%	0%	0%	0%	0%	0%	0%	0%	2%	0%	0%	0%	0%	0%	0%	0%
Microglia_All_Zhang_JNeuro_2014	3%	0%	0%	0%	0%	0%	0%	0%	0%	0%	0%	0%	0%	0%	0%	0%	0%	0%	0%
Mural_All_Zeisel_Science_2015	1%	0%	0%	0%	0%	1%	1%	0%	1%	0%	0%	1%	0%	1%	0%	0%	1%	1%	0%
Mural_Pericyte_Zhang_JNeuro_2014	3%	0%	0%	10%	0%	0%	0%	0%	0%	0%	0%	3%	0%	0%	0%	0%	0%	13%	0%
Mural_Vascular_Daneman_PLOS_2010	4%	0%	0%	6%	0%	0%	0%	0%	0%	0%	2%	2%	0%	2%	0%	0%	2%	2%	0%
Neuron_All_Cahoy_JNeuro_2008	0%	3%	3%	9%	0%	0%	6%	1%	5%	0%	0%	3%	0%	0%	0%	1%	0%	0%	0%
Neuron_All_Darmanis_PNAS_2015	0%	0%	0%	0%	0%	0%	19%	0%	0%	0%	0%	0%	0%	0%	0%	0%	0%	0%	0%
Neuron_All_Zhang_JNeuro_2014	0%	0%	0%	8%	3%	3%	25%	0%	5%	0%	0%	0%	0%	0%	0%	0%	0%	0%	0%
Neuron_CorticoSpinal_Doyle_Cell_2008	0%	4%	0%	16%	0%	0%	0%	0%	0%	0%	0%	0%	0%	0%	0%	0%	0%	0%	0%
Neuron_CorticoStriatal_Doyle_Cell_2008	100%	0%	4%	0%	0%	0%	0%	0%	0%	0%	0%	0%	0%	0%	0%	0%	0%	0%	0%
Neuron_CorticoThalamic_Doyle_Cell_2008	0%	100%	0%	16%	0%	0%	0%	20%	0%	0%	0%	4%	0%	0%	0%	0%	4%	0%	0%
Neuron_Glutamate_Sugino_NatNeuro_2006	1%	0%	100%	4%	0%	0%	0%	1%	0%	0%	0%	3%	0%	0%	0%	3%	0%	0%	0%
Neuron_Pyramidal_Cortical_Zeisel_Science_2015	0%	1%	1%	100%	0%	0%	2%	0%	0%	0%	0%	0%	0%	0%	0%	0%	1%	1%	1%
Neuron_GABA_Sugino_NatNeuro_2006	0%	0%	0%	3%	100%	0%	38%	0%	0%	0%	0%	0%	0%	0%	0%	0%	0%	0%	0%
Neuron_Interneuron_CORT_Doyle_Cell_2008	0%	0%	0%	4%	0%	100%	12%	12%	0%	0%	0%	4%	0%	0%	0%	0%	0%	0%	0%
Neuron_Interneuron_Zeisel_Science_2015	0%	0%	0%	2%	4%	1%	100%	0%	3%	0%	0%	1%	0%	0%	0%	0%	0%	1%	0%
Neuron_Neuron_CCK_Doyle_Cell_2008	0%	8%	4%	0%	0%	12%	0%	100%	0%	0%	0%	4%	0%	0%	0%	0%	0%	0%	0%
Neuron_Neuron_PNOC_Doyle_Cell_2008	0%	0%	0%	4%	0%	0%	46%	0%	100%	0%	0%	0%	0%	0%	0%	0%	4%	0%	0%
Oligodendrocyte_All_Cahoy_JNeuro_2008	0%	0%	0%	0%	0%	0%	0%	0%	0%	100%	4%	48%	12%	6%	20%	2%	0%	0%	0%
Oligodendrocyte_All_Doyle_Cell_2008	0%	0%	0%	0%	0%	0%	0%	0%	0%	8%	100%	4%	0%	0%	0%	4%	0%	8%	0%
Oligodendrocyte_All_Zeisel_Science_2015	0%	0%	0%	0%	0%	0%	0%	0%	0%	5%	0%	100%	2%	2%	4%	2%	0%	0%	0%
Oligodendrocyte_Mature_Darmanis_PNAS_2015	0%	0%	0%	0%	0%	0%	0%	0%	0%	29%	0%	48%	100%	5%	24%	0%	0%	0%	0%
Oligodendrocyte_Mature_Doyle_Cell_2008	0%	0%	0%	0%	0%	0%	0%	0%	0%	12%	0%	40%	4%	100%	8%	0%	0%	4%	0%
Oligodendrocyte_Myelinating_Zhang_JNeuro_2014	0%	0%	0%	0%	0%	0%	0%	0%	0%	25%	0%	48%	13%	5%	100%	0%	0%	0%	0%
Oligodendrocyte_Newly-Formed_Zhang_JNeuro_2014	0%	0%	5%	3%	0%	0%	0%	0%	0%	3%	3%	23%	0%	0%	0%	100%	3%	0%	0%
Oligodendrocyte_Progenitor Cell_Darmanis_PNAS_2015	0%	5%	0%	10%	0%	0%	5%	0%	5%	0%	0%	5%	0%	0%	0%	5%	100%	19%	0%
Oligodendrocyte_Progenitor Cell_Zhang_JNeuro_2014	0%	0%	0%	5%	0%	0%	13%	0%	0%	0%	5%	0%	0%	3%	0%	0%	10%	100%	0%
RBC_All_GeneCardSearch_Hemoglobin_ErythrocyteSpecific	0%	0%	0%	12%	0%	0%	6%	0%	0%	0%	0%	0%	0%	0%	0%	0%	0%	0%	100%

Fig C. Identifying non-specific "cell-type specific genes". Within BrainInABlender, the data from genes that were identifed as being specific to more than one category of cell type (e.g., a gene previously-identified as being "specifically expressed" in both microglia and endothelial cells) were removed before averaging the individual cell type indices within each of the ten primary categories to create the consolidated cell-type indices used throughout our paper. In both figure panels, the primary categories of cell type are color-coded similar to **Fig A. A)** The number of genes identified as cell type specific in each publication in our database vs. the percentage that were actually found to be truly specific to that cell type (i.e., not identified as "specific" to another category of cell type in a different publication). **B)** The percentage overlap between each list of cell type specific genes (row) and all other lists of cell type specific genes (columns). The matrix is color-coded with a gradient from blue (indicating 0% overlap) to red (indicating 100% overlap). The denominator in the percentage overlap equation was the cell type category specified by the row. Note that many of the genes identified as "non-specific" are shared by similar cell type categories (e.g., Oligodendrocytes vs. Oligodendrocyte_Immature), but two gene lists derived from Doyle et al.[15] appeared to include non-specific genes from highly divergent categories: Neuron_CorticoSpinal_Doyle_Cell_2008 strongly overlaps with genes identified as specific to endothelial cells, Neuron_CorticoStriatal_Doyle_Cell_2008 strongly overlaps with genes identified as specific to microglia. In later versions of BrainInABlender, we have removed gene lists derived from Doyle et al.[15] for this reason.

Next, to estimate the limitations and noise inherant in our technique, we also constructed *in silico* mixtures of 100 cells with known percentages of each cell type by randomly sampling from each purified cell dataset (with replacement). We found that the consolidated cell type indices strongly predicted the

percentage of their respective cell type included in our artificial mixtures in a linear manner across a range of values likely to encompass the true proportion of these cells in our cortical samples. The amount of noise present in these predictions varied by data type, with the predictions generated from single-cell data having substantially more noise than those generated from pooled, purified cells, but most of the data (+/- 1 stdev) still fell within +/- 5% of the prediction **(Fig D)**. Therefore, we concluded that cell type indices are a relatively easy manner to predict relative cell type balance across samples.

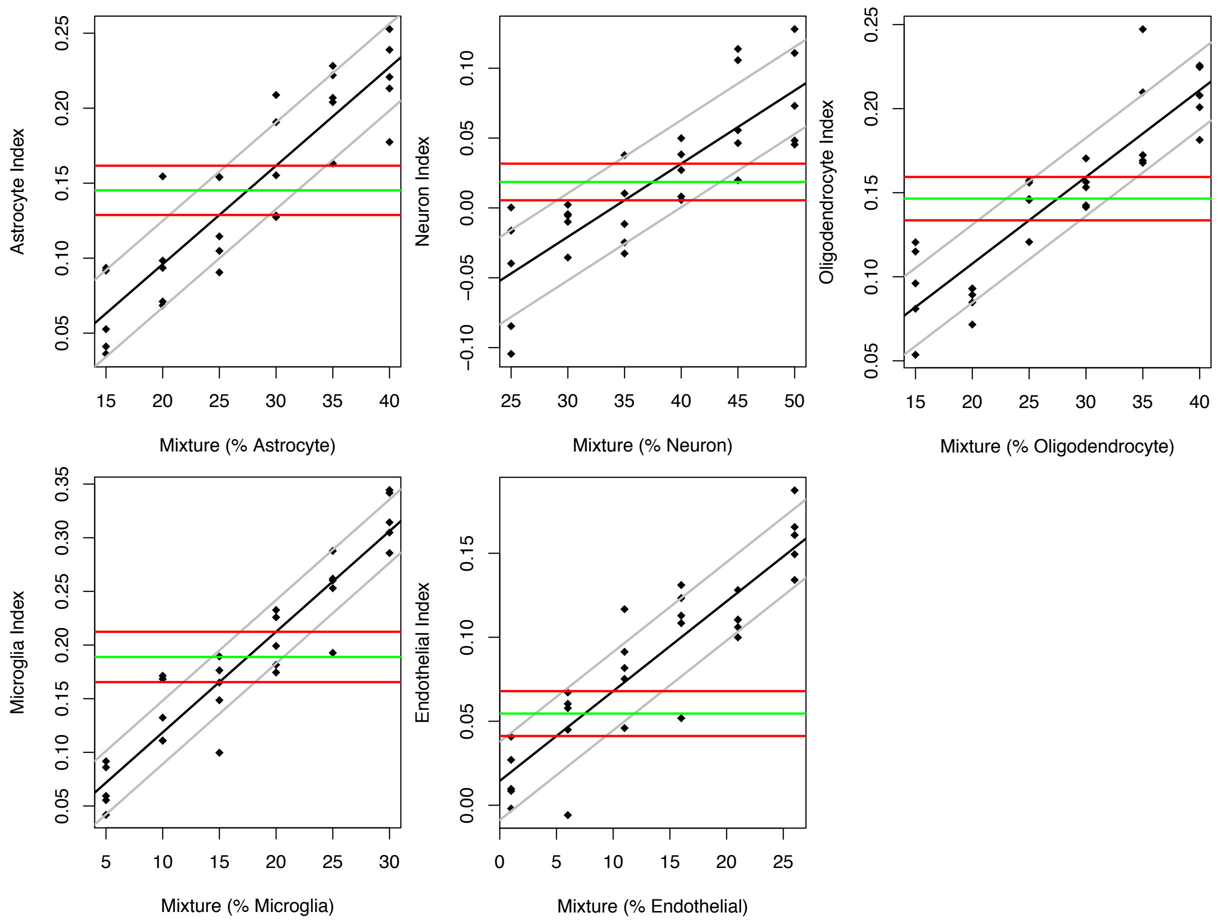

Fig D. Cell type indices successfully predict the percentage of cells of a particular type in artificial mixtures of 100 cells created using single-cell RNA-Seq data. Depicted are the cell type indices (y-axis) calculated for mixed cell samples generated in silico using random sampling (with replacement) from a human single cell RNA-Seq dataset [2]. Each sample contains 100 cells total, with a designated percentage of the cell type of interest (x-axis), with the percentages designed to roughly span what might be found in cortical tissue samples. The black best fit line is accompanied by the standard error of the regression (gray), and the green and red lines are visual guides to help illustrate a 5% increase in the cell type of interest. The results from a similar analysis using the smaller mouse purified, pooled cell type RNA-Seq dataset [18] showed the same trends but with half as much variability.

Comparison of our method vs. PSEA: Predicting cell identity in a single-cell RNA-Seq dataset

Although we generated our method independently to address microarray analysis questions that arose within the Pritzker Neuropsychiatric Consortium, we later discovered that it was quite similar to the technique of population-specific expression analysis (PSEA, [12]) with several notable differences. Similar to our method, PSEA is a carefully-validated analysis method which aims to estimate cell type-differentiated disease effects from microarray data derived from brain tissue of heterogeneous composition and approaches this problem by including the averaged, normalized expression of cell type specific markers within a larger linear model that is used to estimate differential expression in microarray data [10–12]. Analyses using PSEA similarly indicated that individual variability in neuronal, astrocytic, oligodendrocytic, and microglial cell content was sufficient to account for substantial variability in the vast majority of probesets in microarray data from human brain samples, even within non-diseased samples [12]. The differences between our techniques are mostly due to the recent growth of the literature documenting cell type specific expression in brain cell types. PSEA uses a very small set of markers (4-7) to represent each cell type, and screens these markers for tight co-expression within the dataset of interest, since co-expression networks have been previously demonstrated to often represent cell type signatures in the data [94]. This is essential for the analysis of microarray data for brain regions that have not been well characterized for cell type specific expression (e.g., the substantia nigra), but risks the possibility of closely tracking variability in a particular cell function instead of cell content (as described in our results related to aging). Our analysis focused on the well-studied cortex, thus enabling us to utilize hundreds of cell type specific markers derived from a variety of experimental techniques.

Our manner of normalizing data also differs: PSEA normalizes the expression values for each gene by dividing by the average expression of that gene across samples, whereas we use z-score normalization, both at the level of the individual transcript and later at the level of the gene level summary data. Due to

the dependence of PSEA on ratios, genes that have average expression values that are close to zero can end up with normalized values that are extremely high for a handful of samples. For microarray data, this form of normalization should function well because log2 expression values rarely drop below 5. However, within RNA-Seq, counts of zero are common and therefore we suspected that the ratio-form of normalization used by PSEA might not function optimally for this data type.

Therefore, we decided to run a head-to-head comparison of our method and PSEA using the human single-cell RNA-Seq dataset [2]. To make the comparison as interpretable as possible, we used the same list of cell type specific genes for both methods (the genes in our database used to construct BrainInABlender's consolidated cell type indices). In order to avoid circular reasoning, we also excluded any cell type specific genes that had originally been identified by the publication currently used as the test dataset [2]. Then we used the marker() function from the PSEA package to calculate the "Reference Signal" for the most common primary categories of cell types (astrocytes, endothelial cells, microglia, mature oligodendrocytes, and neurons). For our method, we used a procedure similar to BrainInABlender. We applied a z-score transformation to the data for each gene (mean=0, sd=1), and then either averaged by the primary cell type category (to conduct an analysis paralleling PSEA), or averaged the data from the cell type specific genes identified by each publication, followed by averaging by primary cell type category (to create consolidated cell type indices like BrainInABlender).

To compare the efficacy of each method, we ran a linear model to determine the percentage of variation in the population "reference signal" (PSEA) or "cell type index" (our method) accounted for by the cell type identities assigned to each cell in the original publication [2]. We found that both the population reference signals (PSEA) and cell type indices (our method) for each cell were strongly related to their previously-assigned cell type identity, but in general the relationship was stronger when using our method: on average, 34% of the variation in the PSEA reference signal for each cell type was accounted for by cell identity, whereas an average of either 45% or 49% of the variation in our cell type indices was accounted for by cell identity using either the simplified or consolidated versions of our method, respectively (**Fig E part A**). An illustration of this improvement can be found in **Fig E part B**: note the

presence of extreme outliers in the PSEA population reference signal. We conclude that the simple use of a different normalization method is sufficient to make our method more effective at predicting cell type balance in some datasets. We also find that averaging the predictions drawn from the cell type specific genes identified by multiple publications into a consolidated index produces some additional improvement.

A.

Method of deriving a statistical cell type signal:

**Signal from cell type specific genes for:**	**PSEA** (mean signal ratio average)	**Our Cell Type Indices** (z-score average)	**Our Cell Type Indices:** After first averaging by Publication
Astrocytes	34%	52%	57%
Oligodendrocytes	38%	45%	50%
Microglia	36%	42%	51%
Endothelial	30%	28%	33%
Neurons	33%	57%	53%

B.

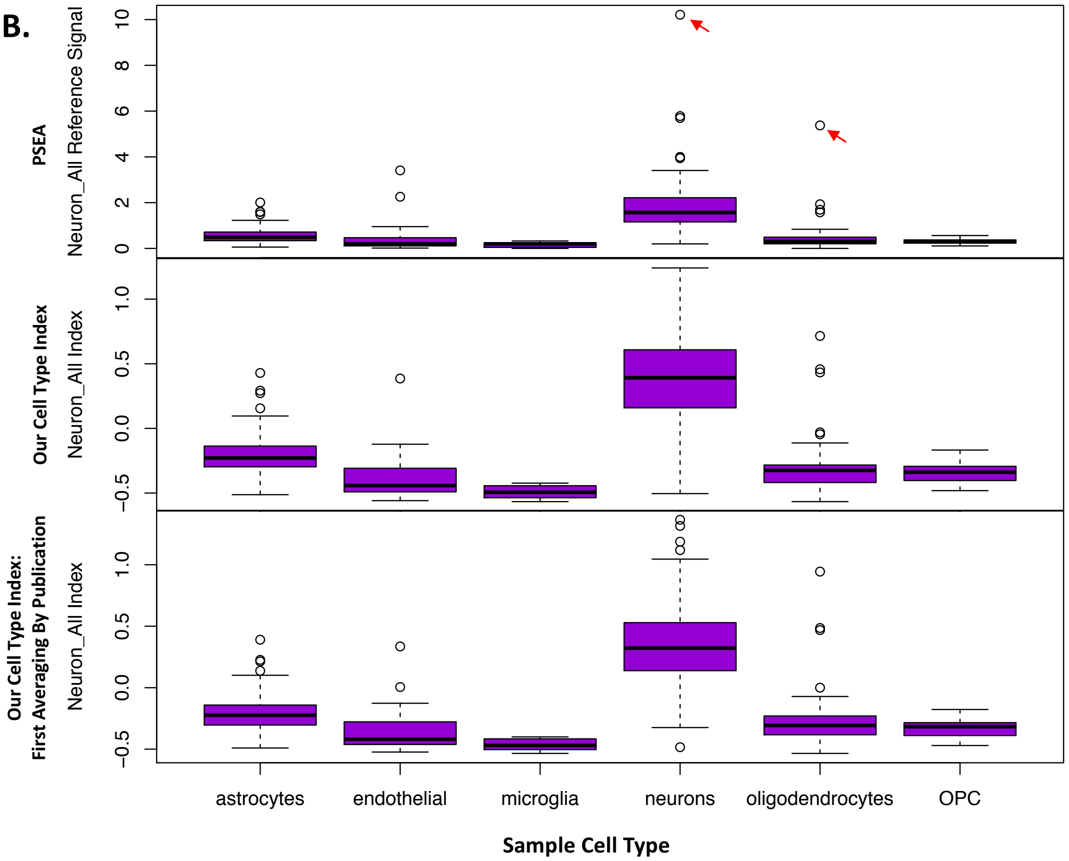

Fig E. The method for deriving predicted relative cell content determines the strength of the relationship with sample cell type. Depicted is a comparison of the efficacy of three different manners of predicting the relative cell content of samples in a human single-cell RNA-seq dataset [2]**:** 1) the "population reference signal" generated by PSEA, 2) a simplified version of our method that is meant to be relatively analogous to PSEA (a simple average of the z-score-transformed data for all genes specific to a particular cell type in our database), 3) the version of our method used in this manuscript, which consolidates the predictions derived from the cell type specific genes identified in different publications. **A.** For each of these methods of predicting relative cell content (columns) the table provides the percentage of variation (R^2) that is accounted for by the original cell type identities of the samples provided by the publication [2] for predictions for each of the major cell types (rows). Overall, there is a strong relationship between the predictions generated by all methods and sample cell type identity, but the method used in this manuscript produces predictions that best fit sample cell type. **B.** As an example, boxplots illustrate the distribution of each of the predictions for neuronal content across samples identified as different cell types in the original publication([2], x-axis). Note the presence of several extreme outliers (red arrows) in the predictions produced by PSEA– a similar pattern was seen for all other cell types.

Using similar methodology, we also calculated the population "reference signal" with PSEA for microarray data from artificially-created mixtures of cultured cells (GSE19380). The results strongly tracked the actual cell content of the mixed samples (**Fig F**) in a manner that was not strikingly better or worse than the predictions made using BrainInABlender (**Fig 6**). This again drives home the fact that the ratio-based normalization methods used in PSEA are particularly incompatible with low count data in RNA-Seq – results derived from microarray data are fine.

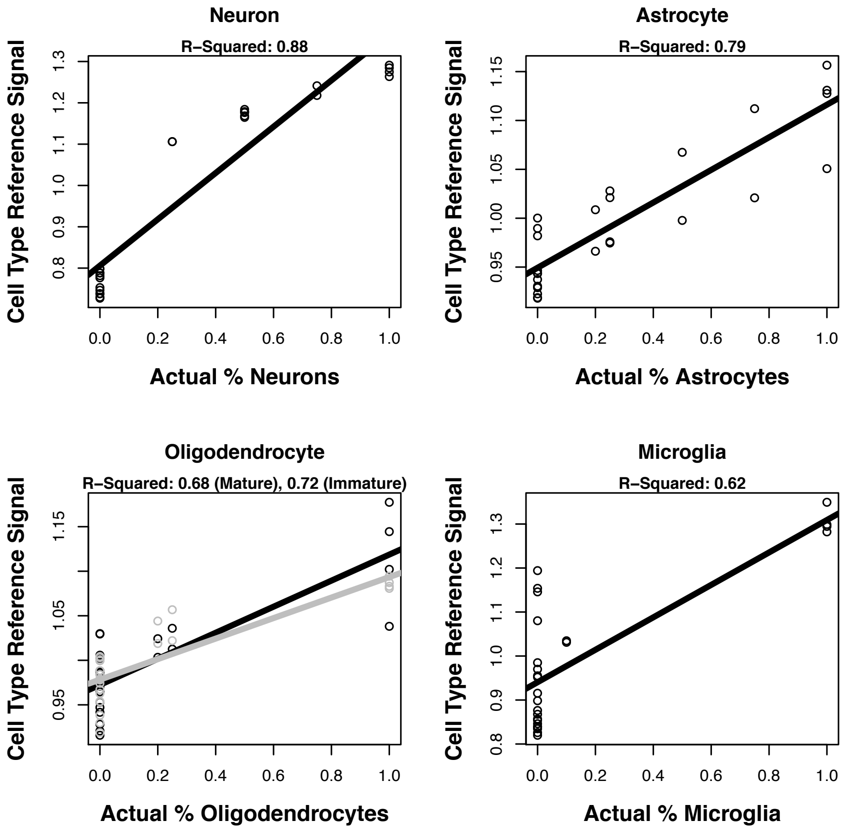

Fig F. Relative cell content predictions made using PSEA and our cell type specific gene lists. Using a microarray dataset derived from samples that contained artificially-generated mixtures of cultured cells (GSE19380; [12]), we found that the relative cell content predictions ("cell type reference signal") produced by PSEA closely reflected actual known content, similar to the predictions made by BrainInABlender (**Fig 6**).

Does the reference dataset matter? There is a strong convergence of cell content predictions derived from cell type specific transcripts identified by different publications

Similar to what we observed during our validation analyses using data from purified cell types, we found that the predicted cell content for the post-mortem human cortical samples ("cell type indices") was similar regardless of the methodology used to generate the cell type specific gene lists used in the predictions. Within all five of the human cortical transcriptomic datasets, there was a strong positive correlation between cell type indices representing the same cell type, even when the predictions were

derived using cell type specific gene lists from different species, cell type purification strategies, and platforms. Clustering within broad cell type categories was clear using visual inspection of the correlation matrices (**Fig G**), hierarchical clustering, or consensus clustering (ConsensusClusterPlus: [95]) and persisted even after removing data from genes identified as cell type specific in multiple publications (*e.g.,* gene expression identified as astrocyte-expression in both Cahoy_Astrocyte and Zhang_Astrocyte; **Fig H**). In some datasets, the cell type indices for support cell subcategories were nicely clustered and in others they were difficult to fully differentiate (**Fig G**). Clustering was not able to reliably discern neuronal subcategories (interneurons, projection neurons) in any dataset. Similar to our previous validation analyses, oligodendrocyte progenitor cell indices derived from different publications did not strongly correlate with each other, perhaps due to heterogeneity in the progenitor cell types sampled by the original publications.

A.

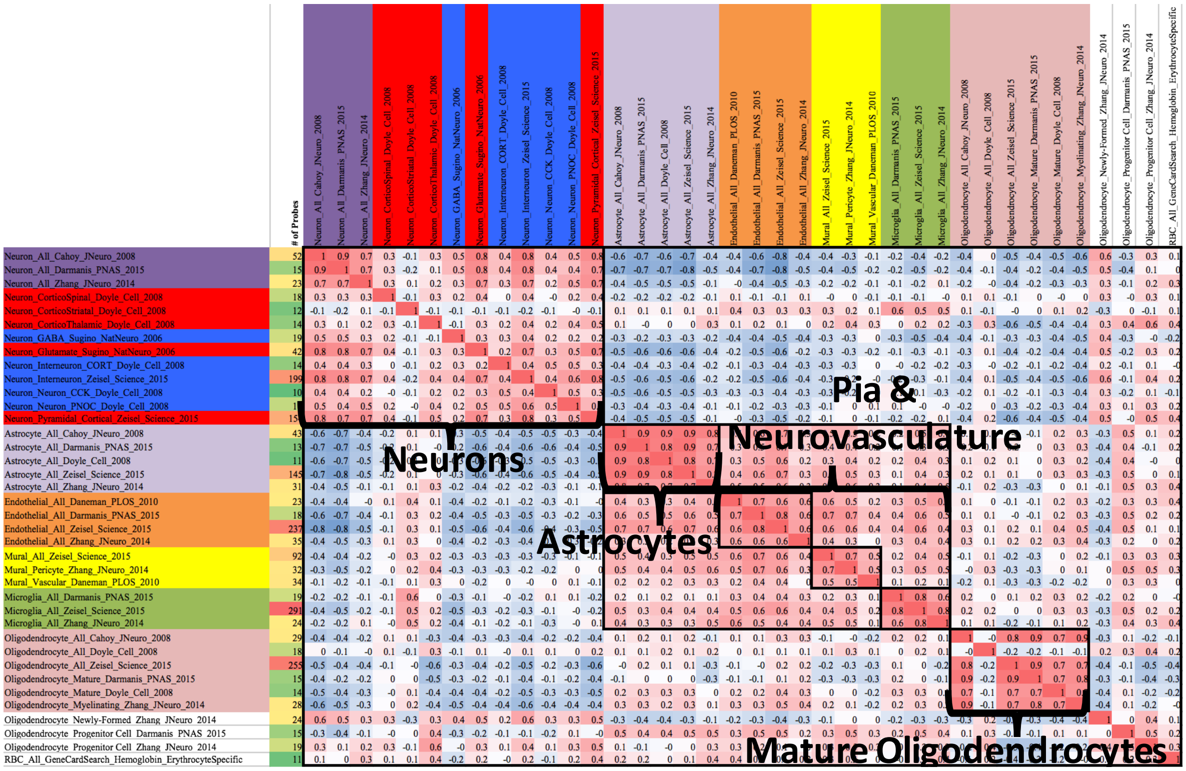

B.

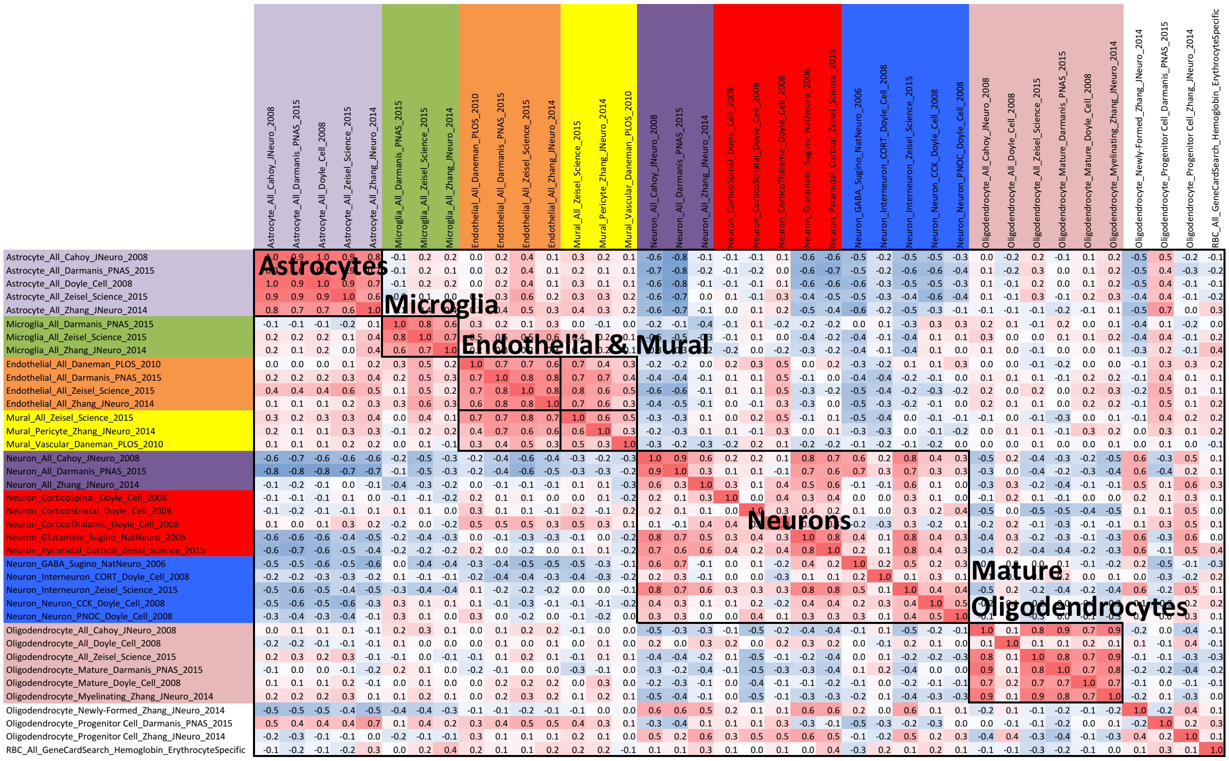
Astrocytes
Microglia
Endothelial & Mural
Neurons
Mature
Oligodendrocytes

C.

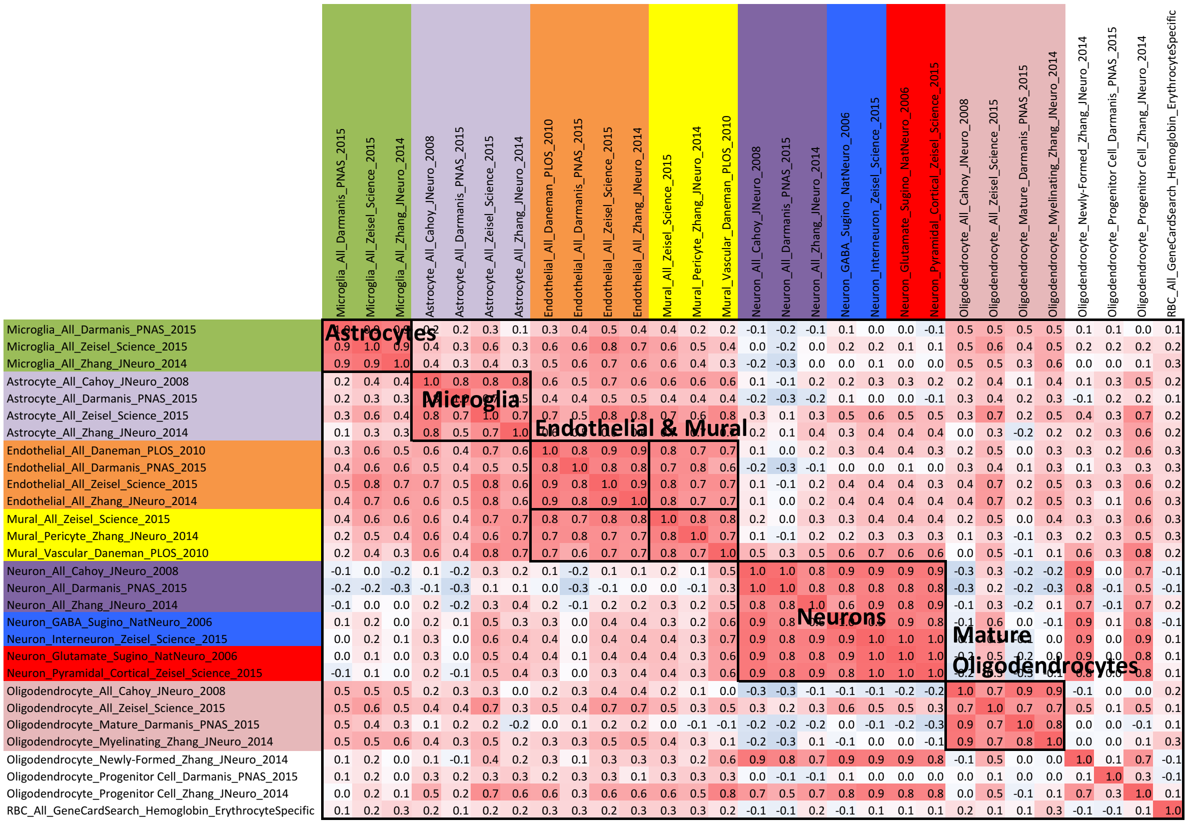
Astrocytes
Microglia
Endothelial & Mural
Neurons
Mature
Oligodendrocytes

Fig G. There is a convergence of cell content predictions derived from cell type specific transcripts identified by different publications. A. The similarity of different cell type indices in the Pritzker cortical dataset can be visualized using a correlation matrix. Within this matrix, correlations can range from a strong negative correlation (-1, blue) to a strong positive correlation (1, red), therefore a large block of pink/red correlations is indicative of cell type indices that tend to be enriched in the same samples. The axis labels for cell type indices representing the same category of cell are color-coded similar to **Fig B**. The number of probes included in each index is present in the far left column (also color-coded, with green indicating few probes and red indicating many probes). **B-C.** Examples of the cell type index correlation matrices from the replication cortical datasets: **B.** Lanz et al. (GSE53987), **C**. CMC RNA-Seq.

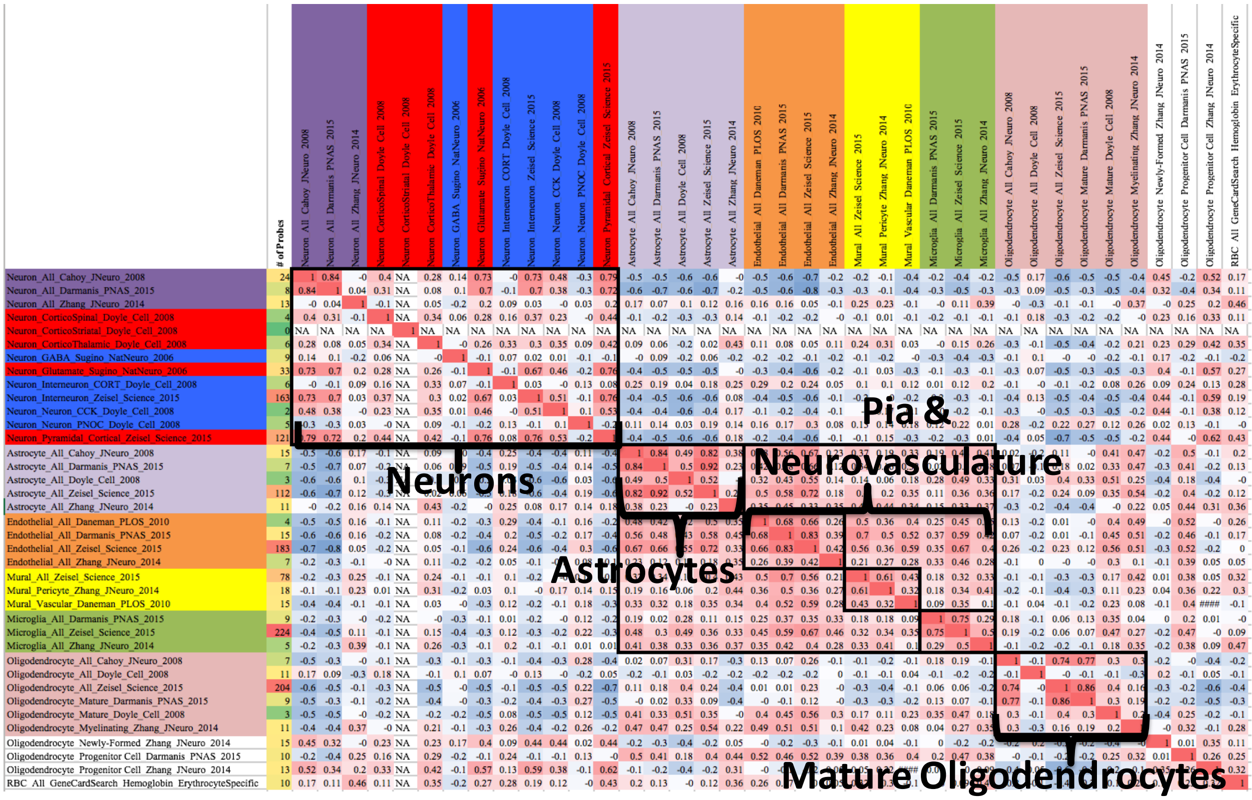

Fig H. The convergence of cell content predictions derived from cell type specific transcripts originating from different publications remains after removing overlapping transcripts. This figure follows the format of **Fig G** (Pritzker dataset), but uses cell type indices calculated following removal of any genes identified as present in more than one index. The similarity of different cell type indices are visualized using a correlation matrix, with color-coded labels for cell type indices representing the same category of cell.

Cell type indices predict other transcripts known to be enriched in specific cell types

To identify other transcripts important to cell type specific functions in the human cortex, we ran a linear model on the signal from each gene probeset in the Pritzker microarray dataset that included each of the ten consolidated primary cell type indices as well as the six traditional co-variates ("Model 5", **Fig 4**). Shown in **Fig I** are the most significant 10 gene probe sets positively associated with each cell type within the model.

Astrocyte	Endothelial	Microglia	Mural	Neuron_All	Neuron_ Projection	Neuron_ Interneuron	Mature Oligodendrocyte	Red Blood Cell (RBC)
**NOTCH2**	**HLA-E**	**AIF1**	**TAGLN**	**VSNL1**	**PDE2A**	**TAC3**	**KLK6**	**HBD**
**SDC2**	**EPAS1**	**LAPTM5**	**MYL9**	**SYT1**	USF2	SLC24A3	**UGT8**	**HBB**
NTRK2	CLCN7	**IRF8**	MYH11	**SYNGR3**	DGKZ	**GAD1**	**MAG**	**PKLR**
**CLDN10**	**CLDN5**	**FCER1G**	CNN1	**NEFL**	**NUAK1**	**KIT**	**ELOVL1**	PGC
**FGFR3**	PAK4	**PTPRC**	MGP	**NRXN1**	SLC38A7	**GAD2**	**EVI2A**	*NA*
**APOE**	MYOF	**LAIR1**	**ACTA2**	**SNAP25**	BEGAIN	**ERBB4**	**PLLP**	DKK4
**EZR**	**ICAM2**	**LY86**	TP53I11	BCL2L1	KIAA0182	**LHX6**	**MOG**	LIPE
**SLC1A3**	ABCB1	FPR1	**COL18A1**	**MAPK1**	**KIF21B**	**SLC6A1**	**ASPA**	SPDEF
CST3	**GPR116**	**C3**	**TPM2**	EEF1A2	**PLXNA1**	**RELN**	**TF**	C19orf57
**MLC1**	**SDPR**	**ALOX5AP**	CRABP1	MEF2C	SLC8A2	ARL4C	**MAL**	*NA*

Fig I. The top 10 transcripts associated with each cell type index include those previously-identified as cell type enriched in the literature. Transcripts are identified by official gene symbol. Yellow labels identify transcripts included in the original cell type index, orange transcripts were previously-identified as cell type enriched in the literature but were not included in the database used to create the index. Please note that not all of the genes listed in the top ten list associated with the Red Blood Cell index would survive a traditional threshold for false detection (q<0.05).

Many of the top gene probesets that we found to be related to each of the cell type indices are already known to be associated with that cell type in previous publications, validating our methodology. Importantly, this is true even when the genes were not included in the original list of cell type specific genes used to generate the index. For example, we found that HLA-E and EPAS1 were both strongly associated with our endothelial index, and both are known to be involved in endothelial cell activation [96][97]. NOTCH2, one of the top astrocyte-related genes, promotes astrocytic cell lineage [98], and

APOE is primarily secreted by astrocytes in the central nervous system [99]. One of the top interneuron genes, LHX6, is specifically enriched in parvalbumin-containing interneurons [2]. Another top interneuron gene, ERBB4, controls the development of GABA circuitry in the cortex [100]. Many top neuron-related genes relate to synaptic function (SYT1, SYNGR3, NRXN1; http://www.genecards.org/). The top projection neuron-related gene, PDE2A, is preferentially expressed in cortical pyramidal neurons [101], and KIF21B is a kinesin found in the dendrites of pyramidal neurons [102]. We also rediscovered probesets representing genes that were listed as alternative orthologs to those included in our original cell type specific gene lists (oligodendrocytes: EVI2A vs.CTD-2370N5.3, microglia: LAIR1 vs. LAIR2, mural cells: COL18A1 vs. COL15A1, ACTA2 vs. ACTG1). Altogether, these results suggest that our cell type indices were associated with the variability of transcripts in the cortex that represented particular cell types and could re-identify known cell type specific markers.

Additional figures and results

Inferred cell type composition explains a large percentage of the sample-sample variability in microarray data from macro-dissected human cortical tissue

Within the four non-Pritzker human cortical tissue datasets, the relationships between the top principal components of variation and the consolidated cell type indices were similarly strong (**Fig J**), even though these datasets had received less preprocessing to remove the effects of technical variation. Within the GSE21935 dataset [31] the first principal component of variation (PC1) accounted for 37% of the variation in the dataset and seemed to represent a gradient running from samples with high predicted support cell content (PC1 vs. endothelial index: R^2=0.85, p<3.6e-18, PC1 vs. astrocyte index: R^2=0.67, p<3.6e-11) to samples with high predicted neuronal content (PC1 vs. neuron_all index: R^2=0.85, p<3.9e-

18). Within GSE53987 [30], which had samples derived exclusively from gray-matter-only dissections, PC1 accounted for 13% of the variation in the dataset and was highly correlated with predicted astrocyte content (PC1 vs. astrocyte index: R^2=0.80, p<4.6e-24). In GSE21138 (39), which also had samples derived exclusively from gray-matter-only dissections, PC1 accounted for 23% of the variation in the dataset and was strongly related to technical variation (batch), but PC2, which accounted for 14% of the variation in the dataset, again represented a gradient from samples with high predicted support cell content to high predicted neuronal content (PC2 vs. astrocyte: R^2=0.56, p<8.3e-11, PC2 vs. neuron_all: R^2=0.54, p<2.3e-10). Finally, within the CMC RNA-Seq dataset, PC1 accounted for 16% of the variation in the dataset and was highly correlated with predicted projection neuron content (PC1 vs. Neuron_Projection: R^2=0.54, p=5.77e-104).

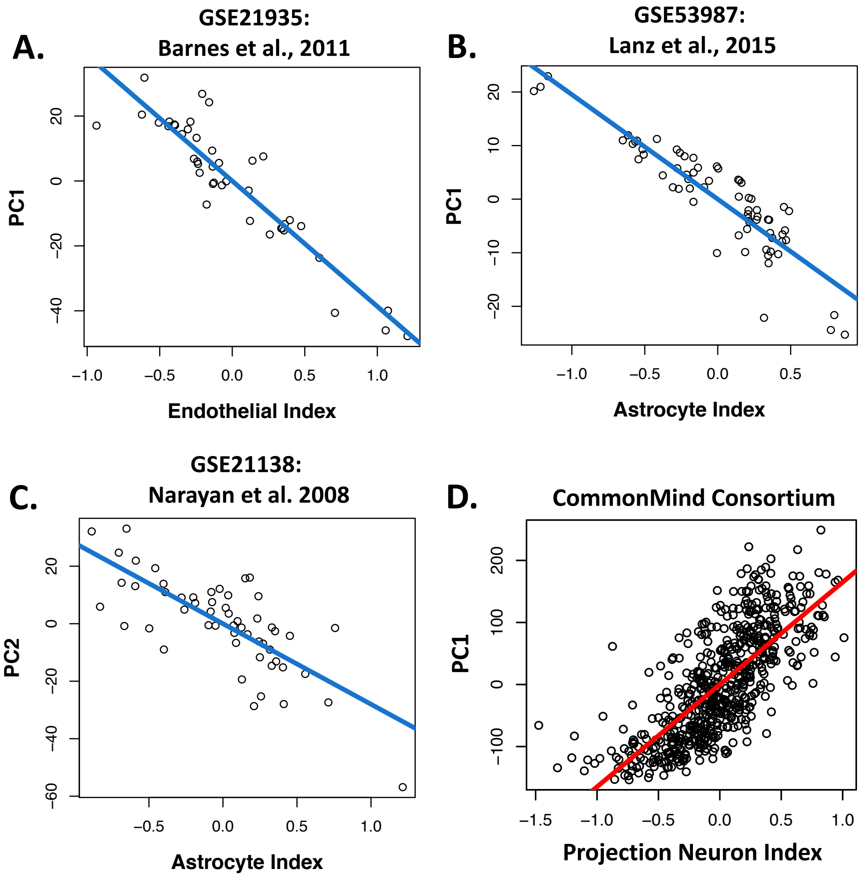

Fig J. Replication: Cell content predictions explain a large percentage of the variability in microarray and RNA-Seq data derived from the human cortex. The results shown above illustrate the strongest relationship between the top principal components of variation (PC1 or PC2) and cell type in

each of the four human replication datasets discussed in the paper: **A)** GSE21935**:** Barnes et al. 2011; **B)** GSE53987: Lanz et al. 2015; **C)** GSE21138: Narayan et al. 2008; **D)** CommonMind Consortium.

When digging deeper, we found that none of the original 38 publication-specific cell type indices were noticeably superior to the consolidated indices when predicting the principal components of variation in the datasets. Human-derived indices did not outperform mouse-derived indices, and indices derived from studies using stricter definitions of cell type specificity (fold enrichment cut-off in **Fig 1**) did not outperform less strict indices.

It is difficult to discriminate between changes in cell type balance and cell-type specific function

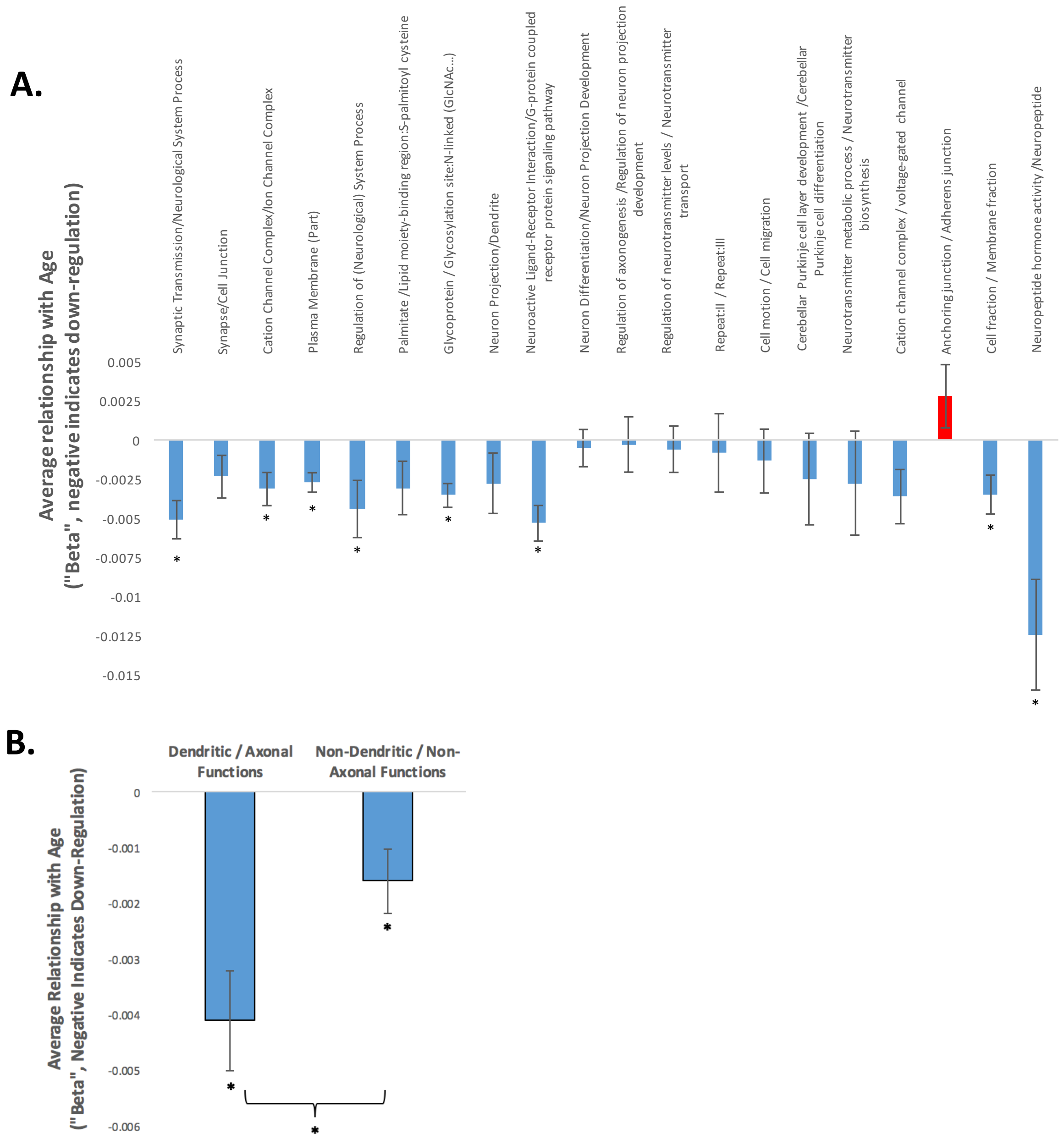

Fig K. The predicted decrease in neuronal cell content in relationship to age is unlikely to be fully explained by synaptic atrophy. Within the list of neuron-specific genes, 240 functional clusters were identified using DAVID. **A)** The genes in 19 out of the top 20 functional clusters showed decreased expression with age on average, as determined within a linear model that controlled for traditional confounds ("Model 2"). Depicted is the average effect of age +/-SE for each cluster (asterisks: $p<0.05$, blue=down-regulation, red=up-regulation). Overall, 76% of all 240 functional clusters showed a negative relationship with age on average (**S4 Table**). **B.)** We blindly chose 29 functional clusters that were clearly

related to dendritic/axonal functions and 41 functional clusters that seemed distinctly unrelated to dendritic/axonal functions. Transcripts from both classifications showed an average decrease in expression with age (p=9.197e-05, p=0.008756, respectively), but the decrease was larger for transcripts associated with dendritic/axonal-related functions (p=0.02339). Depicted is the average effect of age +/- SE for each classification of cluster.

Previously-documented psychiatric effects on cortical gene expression within particular cortical cell types or within macro-dissected prefrontal cortex

Validation Datasets:	# of Genes	Method:	Brain Bank:	# of Subjects	Brain Region	Co-Variates: Controlled?	Co-Variates: Balanced?	Statistical Stringency
Schizophrenia Effects In Particular Cortical Cell Types:								
Reviewed in Lewis & Sweet (2009)	7	ICC/in situ hybridization	Variable, often PITT	Variable	Prefrontal cortex	Variable	Variable	Variable
Arion et al. (2015)	41	LCM-Microarray: Pyramidal Neurons (Layers 3 &5)	PITT	72	BA9	Direction of effect evaluated, but covariates not included in final model.	Sex, Age, PMI, pH, RIN, tissue storage time, race	Top 40 (FDR<0.1 in both layers, Table 2A), Top 2 in Table 2B, FDR<10E-17 for Layer5)
Pietersen et al. (2014)	47	LCM-Microarray: PVALB Interneurons	HBTRC (MacLean)	16	BA42	Batch. Considered effects of Sex, Age, PMI but not included in final model.	Sex, Age, PMI, pH not significantly different	Top 47 (FDR<0.01, FC>2, Table 3)
Mauney et al. (2015)	35	LCM-Microarray: Oligodendrocyte Precursors	HBTRC (MacLean)	18	BA9	None	Sex, Age, PMI, *pH not reported*	Top 35 (FDR<0.001, Table S2)
Psychiatric Effects in Macro-dissected Prefrontal Cortex:								
Mistry et al. (2013)	126	Meta-analysis of microarray data: Schizophrenia effects	Stanley Foundation, HBTRC (MacLean), PITT, CCHPC , MSSM, MHRI	306	BA9, BA10, BA46	Model selection procedure included Batch, Age, pH, Study	Sex, PMI	FDR<0.1 (Table S2)
Choi et al. (2011)	367	Meta-analysis of microarray data: Bipolar effects	Stanley Foundation	83	BA46 (grey matter only)	Batch (Scan Date), pH, Psychosis, Medication at TOD	Age, BMI, *PMI not reported*	FDR<0.05, FC>1.3 (Table S1)

Fig L. Gene lists used to assess whether controlling for cell type while performing differential expression analyses enhances the detection of previously-documented psychiatric effects on cortical gene expression. These lists include genes with documented relationships to psychiatric illness within either 1) particular cortical cell types or 2) macro-dissected cortex. The full lists can be found in **S7 Table.** Abbreviations: LCM: Laser Capture Microscopy, PVALB: Parvalbumin, BA: Brodmann's Area, PMI: Post-mortem interval, FDR: False detection ratio (or q-value), Brain Banks: PITT (University of Pittsburgh), HBTRC (Harvard Brain Resource Tissue Center), CCHPC (Charing Cross Hospital Prospective Collection), MSSM (Mount Sinai Icahn School of Medicine), MHRI (Mental Health Research Institute Australia).

The top diagnosis-related genes identified by models that include cell content predictions pinpoint known risk candidates

Although the inclusion of predicted cell type balance in our model occasionally improved our ability to detect previously-identified relationships with diagnosis, most relationships still went undetected in the Pritzker dataset and none of the diagnosis relationships survived standard p-value corrections for multiple comparisons. This could be due to a variety of factors, including microarray platform and probe sensitivity. Therefore, we decided to ask a complementary question: Of the top diagnosis relationships that we see in our dataset, how many have been previously observed in the literature? If including predicted cell type balance in our models improves the signal to noise ratio of our analyses, then we would expect that the top diagnosis-related genes in our dataset would be more likely to overlap with previous findings. To perform this comparison in an unbiased and efficient manner, we limited our search to PubMed, using as search terms only the respective human gene symbol and diagnosis ("Schizophrenia", "Bipolar", or "Depression"). For the genes related to MDD in our dataset, we also expanded the search to include two highly-correlated traits that are more quantifiable: "Anxiety" and "Suicide". Then we narrowed our results only to studies using human subjects.

Before controlling for cell type, when using a traditional model ("Model#2") we found that only one of the top 10 genes related to diagnosis (FOS: [103,104]) or the presence or absence of psychiatric illness (ALDH1A1: [105]) had been previously noted in the human literature. In contrast, when we used a model that included the five most prevalent cortical cell types (Model#4), we found that five of the top 10 genes associated with Schizophrenia had been previously identified in the literature (ARHGEF2: [106], DOC2A: [107], FBX09: [78], GRM1: [108,109]; CEBPA: [84]), and three of the top 10 genes associated with Bipolar Disorder (ALDH1A1: [105], SNAP25: [110], NRN1:[111]; **Fig M**). This was a significant enrichment in overlap with the literature when compared to 100 randomly-selected genes in the dataset subjected to the same protocol (Schizophrenia: 5/10 vs. 7/100, p=0.0012; Bipolar: 3/10 vs. 8/100, p=0.0610). Likewise, if we replaced diagnosis with a term representing the general presence or absence of

a psychiatric illness, we found that four of the top 10 genes had been previously identified in the literature (ALDH1A1: [105]; HBS1L: [4]; HIVEP2: [112], FBX09: [78], **Fig N**), and 9/10 of the top genes were actually significant with an FDR<0.05 when using permutation based methods (using the R function lmp{lmPerm}, iterations=9999). The top 10 genes associated with psychiatric illness in models selected using forward/backward stepwise model selection (criterion=BIC) similarly included five that had been previously identified in the literature (PRSS16: [113], GRM1: [108,109]; ALDH1A1: [105]; SNAP25: [110]; HIVEP2: [112], a significant improvement in overlap with the literature than what can be seen in 100 randomly-selected genes in the dataset subjected to the same protocol (Fisher's exact test: 5/10 vs.15/100, p=0.0168).

Top Genes Associated with Schizophrenia:

Model 2: **Diagnosis + Confounds**

Gene Symbol	Beta	Pval	FDR
CTRC	-0.13	1.00E-04	4.75E-01
DMP1	-0.06	1.37E-04	4.75E-01
HHLA1	-0.40	1.70E-04	4.75E-01
PITPNB	-0.13	1.96E-04	4.75E-01
DHX32	-0.16	2.61E-04	4.75E-01
ID1	-0.51	2.73E-04	4.75E-01
CRYBB1	-0.12	3.04E-04	4.75E-01
ZNF91	-0.29	3.26E-04	4.75E-01
FAM127B	0.15	3.84E-04	4.75E-01
NPPA	-0.17	3.98E-04	4.75E-01

Model 4: **Diagnosis + 5 Prevalent Cell Types & Confounds**

Gene Symbol	Beta	Pval	FDR
**ARHGEF2**	-0.12	3.96E-05	2.66E-01
**DOC2A**	0.18	4.55E-05	2.66E-01
ID1	-0.53	6.69E-05	2.66E-01
PITPNB	-0.12	8.87E-05	2.66E-01
**FBXO9**	-0.16	2.53E-04	4.48E-01
CTRC	-0.10	3.12E-04	4.48E-01
GPR63	0.12	4.28E-04	4.48E-01
**GRM1**	0.07	4.71E-04	4.48E-01
DHX32	-0.13	4.79E-04	4.48E-01
**CEBPA**	0.15	5.70E-04	4.48E-01

Model 5: **Diagnosis + All Cell Types & Confounds**

Gene Symbol	Beta	Pval	FDR
ID1	-0.54	3.68E-05	2.22E-01
**DOC2A**	0.17	6.26E-05	2.22E-01
**ARHGEF2**	-0.12	6.78E-05	2.22E-01
PITPNB	-0.13	7.41E-05	2.22E-01
**PMP22**	-0.24	1.67E-04	3.64E-01
CRYBB1	-0.10	2.34E-04	3.64E-01
NPPA	-0.14	2.65E-04	3.64E-01
CTRC	-0.10	2.68E-04	3.64E-01
PHLDB1	-0.17	4.41E-04	3.64E-01
**FGFR2**	-0.16	4.49E-04	3.64E-01

Top Genes Associated with Bipolar Disorder:

Model 2: **Diagnosis + Confounds**

Gene Symbol	Beta	Pval	FDR
NDUFS5	-0.15	7.77E-04	1.00E+00
ZNF593	0.16	1.20E-03	1.00E+00
LRRK1	0.10	1.64E-03	1.00E+00
G3BP1	0.13	1.71E-03	1.00E+00
OR7C1	-0.08	1.85E-03	1.00E+00
NARS	-0.08	1.89E-03	1.00E+00
**FOS**	-0.63	2.00E-03	1.00E+00
PITPNB	-0.10	2.22E-03	1.00E+00
GIT2	-0.05	2.44E-03	1.00E+00
UTY	0.04	2.87E-03	1.00E+00

Model 4: **Diagnosis + 5 Prevalent Cell Types & Confounds**

Gene Symbol	Beta	Pval	FDR
**ALDH1A1**	-0.37	7.57E-05	9.06E-01
**SNAP25**	-0.20	3.59E-04	1.00E+00
G3BP1	0.14	7.61E-04	1.00E+00
NDUFS5	-0.15	8.07E-04	1.00E+00
ZNF593	0.16	1.05E-03	1.00E+00
NARS	-0.08	1.07E-03	1.00E+00
CHST1	0.21	1.09E-03	1.00E+00
PITPNB	-0.10	1.11E-03	1.00E+00
TXNDC5	0.14	1.33E-03	1.00E+00
**NRN1**	-0.13	1.42E-03	1.00E+00

Model 5: **Diagnosis + All Cell Types & Confounds**

Gene Symbol	Beta	Pval	FDR
**ALDH1A1**	-0.40	3.05E-05	2.21E-01
**SNAP25**	-0.17	3.69E-05	2.21E-01
CHST1	0.22	4.33E-04	9.98E-01
TRA2A	-0.15	5.78E-04	9.98E-01
G3BP1	0.14	6.58E-04	9.98E-01
ANGEL2	-0.09	7.27E-04	9.98E-01
NARS	-0.08	1.24E-03	9.98E-01
LRRK1	0.10	1.33E-03	9.98E-01
KCTD2	0.10	1.34E-03	9.98E-01
TXNDC5	0.13	1.41E-03	9.98E-01

Top Genes Associated with MDD:

Model 2: **Diagnosis + Confounds**

Gene Symbol	Beta	Pval	FDR
BRD4	0.12	7.10E-05	4.29E-01
PRPH2	0.21	7.16E-05	4.29E-01
MED24	0.15	2.08E-04	7.94E-01
SPRY2	-0.21	3.20E-04	7.94E-01
PRSS16	0.11	3.31E-04	7.94E-01
HEY2	-0.15	6.04E-04	9.16E-01
NEURL	0.11	6.40E-04	9.16E-01
NKAIN1	0.11	1.15E-03	9.16E-01
GGA3	0.09	1.59E-03	9.16E-01
VENTXP1	-0.03	1.60E-03	9.16E-01

Model 4: **Diagnosis + 5 Prevalent Cell Types & Confounds**

Gene Symbol	Beta	Pval	FDR
PRPH2	0.21	6.96E-05	8.34E-01
BRD4	0.11	2.12E-04	9.99E-01
BAP1	0.11	2.77E-04	9.99E-01
PRSS16	0.10	5.10E-04	9.99E-01
ARL4D	-0.13	7.57E-04	9.99E-01
MED24	0.14	7.86E-04	9.99E-01
NKAIN1	0.11	7.97E-04	9.99E-01
REC8	0.12	8.57E-04	9.99E-01
FZD2	0.08	9.54E-04	9.99E-01
KCNN2	-0.14	1.19E-03	9.99E-01

Model 5: **Diagnosis + All Cell Types & Confounds**

Gene Symbol	Beta	Pval	FDR
BRD4	0.12	4.06E-05	4.86E-01
PRPH2	0.20	1.20E-04	5.49E-01
FZD2	0.08	1.37E-04	5.49E-01
BAP1	0.11	4.30E-04	9.99E-01
REC8	0.13	5.80E-04	9.99E-01
ARL4D	-0.13	9.74E-04	9.99E-01
MED24	0.13	1.06E-03	9.99E-01
PRSS16	0.09	1.29E-03	9.99E-01
HBS1L	-0.18	1.33E-03	9.99E-01
NKAIN1	0.10	1.40E-03	9.99E-01

Fig M. When analyzing the full Pritzker dataset, the top genes associated with diagnosis in models that include cell content predictions include genes previously identified in the literature. Depicted are the top 10 genes associated with diagnosis using three different models of increasing complexity, along with their β's (magnitude and direction of effect within the model – blue=downregulation, pink=upregulation), nominal p-values, and p-values that have been corrected for false detection rate (FDR or q-value). Gene symbols that are bolded and highlighted yellow have been previously detected in the human literature in association with their respective diagnosis in papers identified using the PubMed search terms "Schizophrenia" (Row 1) and "Bipolar" (Row 2). None of the top genes associated with major depressive disorder in any of the three models were found to be associated with "Depression", "Anxiety", or "Suicide" on PubMed (Row 3).

Top Genes Associated with Psychiatric Illness:

Model 2: **Psychiatric + Confounds**

Gene Symbol	Beta	Pval	FDR
CLIP2	0.16	2.18E-04	8.71E-01
FAM127B	0.11	2.29E-04	8.71E-01
MED24	0.11	3.91E-04	8.71E-01
R3HDM2	-0.16	4.43E-04	8.71E-01
MAP7D1	0.11	4.61E-04	8.71E-01
**ALDH1A1**	-0.30	5.34E-04	8.71E-01
PITPNB	-0.08	6.38E-04	8.71E-01
FANCC	-0.08	7.08E-04	8.71E-01
TTC31	0.08	8.57E-04	8.71E-01
BTG2	-0.16	8.87E-04	8.71E-01

Model 4: **Psychiatric + 5 Prevalent Cell Types & Confounds**

Gene Symbol	Beta	Pval	FDR
ARL4D	-0.12	1.26E-04	6.37E-01
**ALDH1A1**	-0.24	3.02E-04	6.37E-01
CLIP2	0.14	4.06E-04	6.37E-01
**HBS1L**	-0.16	4.21E-04	6.37E-01
MICALL2	0.09	4.57E-04	6.37E-01
PITPNB	-0.08	4.69E-04	6.37E-01
DNAJB2	0.12	4.80E-04	6.37E-01
PPP6C	-0.07	5.75E-04	6.37E-01
**HIVEP2**	-0.12	5.91E-04	6.37E-01
**FBXO9**	-0.10	7.58E-04	6.37E-01

Model 5: **Psychiatric + All Cell Types & Confounds**

Gene Symbol	Beta	Pval	FDR
ARL4D	-0.12	9.91E-05	5.12E-01
MICALL2	0.08	2.07E-04	5.12E-01
**HIVEP2**	-0.11	2.41E-04	5.12E-01
**SNAP25**	-0.10	3.72E-04	5.12E-01
TRA2A	-0.11	3.79E-04	5.12E-01
CLIP2	0.14	3.79E-04	5.12E-01
FZD2	0.06	4.06E-04	5.12E-01
**ALDH1A1**	-0.22	4.33E-04	5.12E-01
SMARCD3	0.12	4.46E-04	5.12E-01
CHST1	0.16	4.47E-04	5.12E-01

Stepwise Regression: **Top Genes Associated with Psychiatric Illness**

Gene Symbol	Beta	Pval
MED24	0.13	1.83E-05
CLIP2	0.17	4.74E-05
**PRSS16**	0.10	8.86E-05
**GRM1**	0.05	1.11E-04
**ALDH1A1**	-0.23	1.28E-04
ARL4D	-0.11	1.37E-04
**SNAP25**	-0.11	1.39E-04
CHST1	0.16	1.45E-04
**HIVEP2**	-0.12	1.53E-04
TTC31	0.08	1.67E-04

Stepwise Regression: **Top Genes Associated with Suicide**

Gene Symbol	Beta	Pval
DGKE	0.035	1.81E-05
UNKL	0.106	2.40E-05
C11orf95	0.17	6.56E-05
TUBB6	0.162	9.41E-05
NEK3	-0.08	1.72E-04
ZNF592	0.158	2.27E-04
FAM98A	-0.11	3.00E-04
SPSB1	0.087	3.01E-04
CEBPB	0.249	4.04E-04
CHST11	0.069	4.18E-04

Fig N. When analyzing the full dataset, the top genes associated with psychiatric illness in models that include cell content predictions include genes previously identified in the literature. Depicted are the top 10 genes associated with psychiatric illness using three different models of increasing complexity, or associated with psychiatric illness or suicide in models chosen using stepwise regression. Notably, the results from stepwise regression for the diagnosis term are not included in this figure because the term was only included in the model for eight genes total (DHX32, ID1, CSRP1, AKR1B10, TBPL1, HIST1H4F, SETD3, GAL). Formatting follows that of **Fig M**. Note that the p-values associated with stepwise regression are likely to be optimistic due to overfitting. Gene symbols that are bolded and highlighted yellow have been previously detected in the human literature using the PubMed search terms “Schizophrenia”, “Bipolar”, “Depression”, “Anxiety”, or “Suicide”.

Together, we conclude that including cell content predictions in the analysis of macro-dissected microarray data can sometimes improve the sensitivity of the assay for detecting altered gene expression in relationship to psychiatric illness, especially if the dataset is confounded with dissection variation.

Supplementary references

87. Irizarry RA, Hobbs B, Collin F, Beazer-Barclay YD, Antonellis KJ, Scherf U, et al. Exploration, normalization, and summaries of high density oligonucleotide array probe level data. Biostat Oxf Engl. 2003;4: 249–264. doi:10.1093/biostatistics/4.2.249

88. American Psychiatric Association. Diagnostic and Statistical Manual of Mental Disorders (DSM-IV-TR). 4th ed. Washington, D.C.: American Psychiatric Association; 2000.

89. Dai M, Wang P, Boyd AD, Kostov G, Athey B, Jones EG, et al. Evolving gene/transcript definitions significantly alter the interpretation of GeneChip data. Nucleic Acids Res. 2005;33: e175. doi:10.1093/nar/gni179

90. Allen Brain Atlas. Technical White Paper: Microarray Data Normalization, v.1 [Internet]. 2013. Available: help.brain-map.org

91. Davis S, Meltzer PS. GEOquery: a bridge between the Gene Expression Omnibus (GEO) and BioConductor. Bioinforma Oxf Engl. 2007;23: 1846–1847. doi:10.1093/bioinformatics/btm254

92. Kim D, Langmead B, Salzberg SL. HISAT: a fast spliced aligner with low memory requirements. Nat Methods. 2015;12: 357–360. doi:10.1038/nmeth.3317

93. Law CW, Chen Y, Shi W, Smyth GK. voom: Precision weights unlock linear model analysis tools for RNA-seq read counts. Genome Biol. 2014;15: R29. doi:10.1186/gb-2014-15-2-r29

94. Oldham MC, Konopka G, Iwamoto K, Langfelder P, Kato T, Horvath S, et al. Functional organization of the transcriptome in human brain. Nat Neurosci. 2008;11: 1271–1282. doi:10.1038/nn.2207

95. Wilkerson MD, Hayes DN. ConsensusClusterPlus: a class discovery tool with confidence assessments and item tracking. Bioinforma Oxf Engl. 2010;26: 1572–1573. doi:10.1093/bioinformatics/btq170

96. Coupel S, Moreau A, Hamidou M, Horejsi V, Soulillou J-P, Charreau B. Expression and release of soluble HLA-E is an immunoregulatory feature of endothelial cell activation. Blood. 2007;109: 2806–2814. doi:10.1182/blood-2006-06-030213

97. Tian H, McKnight SL, Russell DW. Endothelial PAS domain protein 1 (EPAS1), a transcription factor selectively expressed in endothelial cells. Genes Dev. 1997;11: 72–82.

98. Tchorz JS, Tome M, Cloëtta D, Sivasankaran B, Grzmil M, Huber RM, et al. Constitutive Notch2 signaling in neural stem cells promotes tumorigenic features and astroglial lineage entry. Cell Death Dis. 2012;3: e325. doi:10.1038/cddis.2012.65

99. Boyles JK, Pitas RE, Wilson E, Mahley RW, Taylor JM. Apolipoprotein E associated with astrocytic glia of the central nervous system and with nonmyelinating glia of the peripheral nervous system. J Clin Invest. 1985;76: 1501–1513. doi:10.1172/JCI112130

100. Fazzari P, Paternain AV, Valiente M, Pla R, Luján R, Lloyd K, et al. Control of cortical GABA circuitry development by Nrg1 and ErbB4 signalling. Nature. 2010;464: 1376–1380. doi:10.1038/nature08928

101. Stephenson DT, Coskran TM, Kelly MP, Kleiman RJ, Morton D, O'Neill SM, et al. The distribution of phosphodiesterase 2A in the rat brain. Neuroscience. 2012;226: 145–155. doi:10.1016/j.neuroscience.2012.09.011

102. Marszalek JR, Weiner JA, Farlow SJ, Chun J, Goldstein LS. Novel dendritic kinesin sorting identified by different process targeting of two related kinesins: KIF21A and KIF21B. J Cell Biol. 1999;145: 469–479.

103. Rao JS, Harry GJ, Rapoport SI, Kim HW. Increased excitotoxicity and neuroinflammatory markers in postmortem frontal cortex from bipolar disorder patients. Mol Psychiatry. 2010;15: 384–392. doi:10.1038/mp.2009.47

104. Spiliotaki M, Salpeas V, Malitas P, Alevizos V, Moutsatsou P. Altered glucocorticoid receptor signaling cascade in lymphocytes of bipolar disorder patients. Psychoneuroendocrinology. 2006;31: 748–760. doi:10.1016/j.psyneuen.2006.02.006

105. Le-Niculescu H, Patel SD, Bhat M, Kuczenski R, Faraone SV, Tsuang MT, et al. Convergent functional genomics of genome-wide association data for bipolar disorder: comprehensive identification of candidate genes, pathways and mechanisms. Am J Med Genet Part B Neuropsychiatr Genet Off Publ Int Soc Psychiatr Genet. 2009;150B: 155–181. doi:10.1002/ajmg.b.30887

106. Konopaske GT, Subburaju S, Coyle JT, Benes FM. Altered prefrontal cortical MARCKS and PPP1R9A mRNA expression in schizophrenia and bipolar disorder. Schizophr Res. 2015;164: 100–108. doi:10.1016/j.schres.2015.02.005

107. Glessner JT, Reilly MP, Kim CE, Takahashi N, Albano A, Hou C, et al. Strong synaptic transmission impact by copy number variations in schizophrenia. Proc Natl Acad Sci U S A. 2010;107: 10584–10589. doi:10.1073/pnas.1000274107

108. Ayoub MA, Angelicheva D, Vile D, Chandler D, Morar B, Cavanaugh JA, et al. Deleterious GRM1 mutations in schizophrenia. PloS One. 2012;7: e32849. doi:10.1371/journal.pone.0032849

109. Frank RAW, McRae AF, Pocklington AJ, van de Lagemaat LN, Navarro P, Croning MDR, et al. Clustered coding variants in the glutamate receptor complexes of individuals with schizophrenia and bipolar disorder. PloS One. 2011;6: e19011. doi:10.1371/journal.pone.0019011

110. Etain B, Dumaine A, Mathieu F, Chevalier F, Henry C, Kahn J-P, et al. A SNAP25 promoter variant is associated with early-onset bipolar disorder and a high expression level in brain. Mol Psychiatry. 2010;15: 748–755. doi:10.1038/mp.2008.148

111. Fatjó-Vilas M, Prats C, Pomarol-Clotet E, Lázaro L, Moreno C, González-Ortega I, et al. Involvement of NRN1 gene in schizophrenia-spectrum and bipolar disorders and its impact on age at onset and cognitive functioning. World J Biol Psychiatry Off J World Fed Soc Biol Psychiatry. 2016;17: 129–139. doi:10.3109/15622975.2015.1093658

112. Volk DW, Chitrapu A, Edelson JR, Roman KM, Moroco AE, Lewis DA. Molecular mechanisms and timing of cortical immune activation in schizophrenia. Am J Psychiatry. 2015;172: 1112–1121. doi:10.1176/appi.ajp.2015.15010019

113. Girgenti MJ, LoTurco JJ, Maher BJ. ZNF804a regulates expression of the schizophrenia-associated genes PRSS16, COMT, PDE4B, and DRD2. PloS One. 2012;7: e32404. doi:10.1371/journal.pone.0032404
